# *In Silico* Targeting of Fascin Protein for Cancer Therapy: Benchmarking, Virtual Screening and Molecular Dynamics Approaches

**DOI:** 10.3390/molecules28031296

**Published:** 2023-01-29

**Authors:** Heba H. A. Hassan, Muhammad I. Ismail, Mohammed A. S. Abourehab, Frank M. Boeckler, Tamer M. Ibrahim, Reem K. Arafa

**Affiliations:** 1 Drug Design and Discovery Laboratory, Zewail City of Science and Technology, October Gardens, 6th of October City, Giza 12578, Egypt; 2 Department of Pharmaceutical Chemistry, Faculty of Pharmacy, The British University in Egypt, Al-Sherouk City, Cairo-Suez Desert Road, Cairo 11837, Egypt; 3 Department of Pharmaceutics, College of Pharmacy, Umm Al-Qura University, Makkah 21955, Saudi Arabia; 4 Lab for Molecular Design and Pharmaceutical Biophysics, Department of Pharmacy and Biochemistry, Institute of Pharmaceutical Sciences, University of Tübingen, Auf der Morgenstelle 8, 72076 Tübingen, Germany; 5 Department of Pharmaceutical Chemistry, Faculty of Pharmacy, Kafrelsheikh University, Kafrelsheikh 33516, Egypt; 6 Biomedical Sciences Program, University of Science and Technology, Zewail City of Science and Technology, October Gardens, 6th of October City, Giza 12578, Egypt

**Keywords:** cancer, Fascin, docking, virtual screening (VS), benchmarking, DEKOIS 2.0

## Abstract

Fascin is an actin-bundling protein overexpressed in various invasive metastatic carcinomas through promoting cell migration and invasion. Therefore, blocking Fascin binding sites is considered a vital target for antimetastatic drugs. This inspired us to find new Fascin binding site blockers. First, we built an active compound set by collecting reported small molecules binding to Fascin’s binding site 2. Consequently, a high-quality decoys set was generated employing DEKOIS 2.0 protocol to be applied in conducting the benchmarking analysis against the selected Fascin structures. Four docking programs, MOE, AutoDock Vina, VinaXB, and PLANTS were evaluated in the benchmarking study. All tools indicated better-than-random performance reflected by their pROC-AUC values against the Fascin crystal structure (PDB: ID 6I18). Interestingly, PLANTS exhibited the best screening performance and recognized potent actives at early enrichment. Accordingly, PLANTS was utilized in the prospective virtual screening effort for repurposing FDA-approved drugs (DrugBank database) and natural products (NANPDB). Further assessment via molecular dynamics simulations for 100 ns endorsed Remdesivir (DrugBank) and NANPDB3 (NANPDB) as potential binders to Fascin binding site 2. In conclusion, this study delivers a model for implementing a customized DEKOIS 2.0 benchmark set to enhance the VS success rate against new potential targets for cancer therapies.

## 1. Introduction

Tumor metastasis is among the main reasons for mortality, accounting for almost 90% of cancer-related deaths [1]. The vital features of metastatic cancer cells are cell invasion and cell migration, involving the reconstruction of the actin cytoskeleton by triggering the formation of protrusive tissue that leads to enhanced motility of tumor cells in various transformed cells, such as lamellipodia, filopodia and invadopodia [2,3,4].

Fascin1 (termed Fascin thereafter) is an actin-bundling protein (F-actin) playing a main role in formation of protrusions of the cell surface by crosslinking actin filaments tightly and making parallel bundles that promote cell migration [5,6]. Therefore, it is absent or down-regulated in normal adult epithelial tissues [7] and overexpressed in various carcinomas which are positively correlated with invasion, metastasis, and poor prognosis [6]. Conversely, cancer cell invasion is reduced by genetic knockdown of Fascin in vitro and in vivo as well [8]. Consequently, many studies have considered Fascin as a potential diagnostic biomarker and a viable therapeutic target for severe carcinomas [8,9].

Fascin was discovered first in sea urchins as an actin-bundling protein [10] and then identified in Drosophila [11], mice [12], Xenopus [13], and humans [14]. Fascin is a 55 kDa monomeric protein doing its function at its monomeric state, however, most actin bundler proteins work as dimers [15]. It consists of four β-trefoil domains, and each one has six two-stranded β-hairpins with a three-fold symmetric orientation [16,17]. Each β-trefoil domain is situated in the cater-corner forming a quadrilateral-like shape [15].

Previous studies proposed that the three distinct surfaces of Fascin have the main role of its actin-bundling activity; the larger binding sites 1 and 2 are in the cavities between β-trefoils 1 and 4, and β-trefoils 1 and 2, respectively, whereas the smaller third site is located in β-trefoil 3 [18,19]. It is suggested that actin-binding site 2 in the connection between β-trefoils 1 and 2 is important for Fascin actin-bundling activity [20].

Moreover, an X-ray crystallography study of mutant Fascin revealed that its actin-binding sites have a coordinated relationship. A mutation occurring in one actin-binding site damages the function of another one due to a concerted conformational change that occurs, thus displaying the inactive configuration of Fascin [18].

Therefore, blocking of the actin-Fascin interaction has been considered an ideal target for cancer treatment. To discover novel Fascin inhibitors, recent studies have identified many molecules working as inhibitors from chemical databases which are still under investigation for biochemical and pathological research [21,22,23,24,25,26,27,28]. Recently, lead compounds have been found to inhibit Fascin efficiently, such as migrastatin (MGS) and its macroketone analogues, N-(1-(4-(trifluoromethyl) benzyl)-1H-indazol-3-yl) furan-2-carboxamide (G2), and its analogues, such as NP-G2-044 and NP-G2-029 [21,22,23,24]. These G2 analogues have been tested in vitro and in vivo, showing significant effects against Fascin, and suppressing the migration ability of breast tumor cells in humans [23,24]. Moreover, other studies performed virtual screening (VS) efforts for discovering novel Fascin inhibitors. Some FDA-approved drugs, such as the antidepressant Imipramine and the antiviral Raltegravir, showed anti-migratory and anti-invasive effects [25,26]. Another study reported Fascin structures co-crystalized with discovered ligands as inhibitors, such as BDP-13176 (PDB ID: 6I18) [27].

Recently, computer-aided drug discovery (CADD) approaches, especially structure-based virtual screening (SBVS), have been commonly applied to new drug discovery in various disciplines [29,30,31,32]. Molecular docking tools become beneficial in predicting the binding poses of novel bioactive compounds and ranking them according to their scoring functions. They can provide data on the binding site of the target protein, helping to assess databases of a huge number of compounds and providing the best binding compounds before being selected for biological screening [33,34,35]. With this approach, the drug research cost gets reduced markedly [36]. Moreover, selection of the appropriate molecular docking tool and assessing its screening performance should be evaluated using benchmarking molecular sets enriching the known active candidates with an inactive compounds set identified as the decoys set. Benchmarking is helpful in decreasing wasted effort and time on an ineffective VS workflow [37,38].

The present study aims at providing a benchmarking-based VS pipeline. Therefore, we started with compiling a bioactive set for the Fascin protein, then generated a challenging decoy set employing DEKOIS 2.0 protocol. Furthermore, a protein structure analysis was conducted to extract a protein model for benchmarking and subsequent VS and MD efforts. Subsequently, we performed a benchmarking analysis for four diverse-in-architecture and popular docking tools, namely: MOE v.20.19.01, AutoDock Vina v.1.1.2, VinaXB, and PLANTS v.1.2, representing free and commercial packages, to propose a tool with high predictive power against Fascin. Accordingly, the best performing tool was employed for the prospective VS campaign against the Fascin protein utilizing a repurposing strategy of FDA-approved drugs and North African Natural Products. The proposed hits were further validated *in silico* via MD simulations and respective analyses.

## 2. Results and Discussion

### 2.1. Selection of Fascin Actives for Decoys Generation

As an initial step, all available Fascin inhibitors were collected from the literature, then the bioactive molecules were manually curated. Due to the novelty of the protein target, many compounds were reported to be in a micromolar range of activity. Therefore, we decided to include them all, and only exclude the one with no determined affinity/activity having Kd values > 100 (µM). Our focus is on those molecules that bind to actin-binding site 2. This ended up with collecting 25 bioactive molecules as shown in Table 1. This low count of known inhibitors for Fascin reflects the needed efforts to enrich the chemical space of its inhibitory activity. Therefore, in the current study, a VS workflow is proposed to enhance the success rate of drug discovery against Fascin. The core scaffolds of the curated active set represent different chemotype classes, namely: Indazole, *N*-phenylacetamide, pyrazolo[3,4-d]pyrimidinone, isoquinolone, naphthyridone, pyrazolo[4,3-c]pyridine, and pyridone [23,24,27]. A summary of relevant data for the active set is listed in Table 1.

### 2.2. Selection of Representative PDB Structure(s) for Fascin

**Figure 1:**
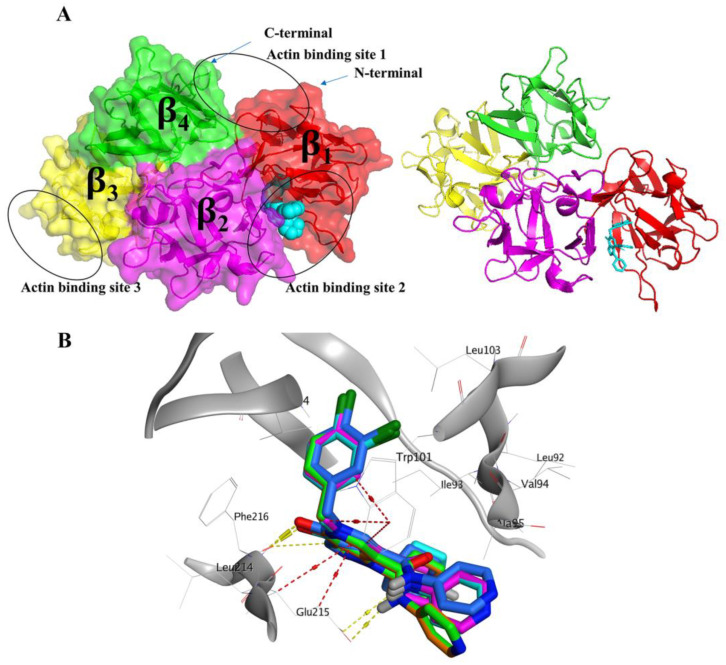
(**A**) The structure of the Fascin-inhibitor complex (PDB ID: 6I18). The Fascin β-trefoil domains 1, 2, 3, and 4 are represented by red, magenta, yellow and green, respectively showing the three actin-binding sites. (**B**) Pose-retrieval docking experiments for the co-crystal ligand (cyan) (PDB: 6I18) using the four docking tools: MOE (magenta), Autodock Vina (green), VinaXB (orange), and PLANTS (blue).

The structure of human Fascin includes 493-amino acids. Its four consecutive β-trefoil domains are formed by the amino acid residues 8–139, 140–260, 261–381, and 382–493, respectively [18,39]. The actin-binding site 1, formed by residues from the N and C termini, is located between β-trefoil 1 and 4. Ser39 amino acid, a highly conserved residue in this area, can be phosphorylated by protein kinase C (PKC). The actin-binding site 2 includes the residues of β-trefoil 1 and 2 lying at the cleft created by them, while actin-binding site 3 is a potential site where β-trefoil 3 is located [18], as shown in Figure 1.

For the selection of a protein structure to be used in the benchmarking study, we searched all Fascin1 structures and downloaded them from the Protein Data Bank (Table S1 in Supplementary Materials). Previous studies revealed that Fascin inhibitors cause a conformational change in Fascin through an induced-fit inhibitory mechanism disrupting the actin-binding sites, and hence impairing its actin-bundling role [24]. Therefore, to account for the protein’s different conformations, their structures were superposed showing their differences in the backbones as indicated by their RMSD values (see Figure S1 in Supplementary Materials). We focused especially on the Fascin structures co-crystallized with ligands in their binding site to detect any structural changes occurring during the ligand–protein binding. Consequently, based on analysis, both Fascin structures (PDB ID: 6I18) and (PDB ID: 6I0Z) were selected to represent two main liganded clusters of conformations. This is indicated by their pairwise RMSD values considering the whole protein structure and their pockets, as demonstrated in Figure S2.

We aim in this study to tackle the virtual screening performance of examples from diverse docking tools, whether publicly available (e.g., AutoDock Vina, VinaXB, and PLANTS), or commercial ones (e.g., MOE). These docking tools represent different architectures in the development of their optimization/search algorithms and scoring functions. For instance, AutoDock Vina is based on the Broyden–Fletcher–Goldfarb–Shanno (BFGS) method for the local optimization and uses its own Vina scoring function [40], while PLANTS employs the Protein–Ligand ANT System algorithm and PLANTS_CHEMPLP_ scorning function [41]. Since the majority of the bioactive compounds for the Fascin protein are halogenated compounds (Table 1), this directed us to utilize a docking tool with a halogen-bonding implementation in the scoring function, such as VinaXB [42], where it was developed based on AutoDock Vina. Moreover, applying a commercial package, we used MOE which employs the London dG scoring function. Nonetheless, the benchmarking analysis can be extended to any other docking tool. Initially, these docking tools were utilized for pose-retrieval docking experiments for the co-crystal ligand (PDB: 6I18). Interestingly, they reflected acceptable results with RMSD values < 2 Å, as shown in Figure 1B.

### 2.3. Benchmarking

To provide valid benchmarking sets for the effective performance of the structure-based VS, prerequisites should be met. First, a set of chosen and well-described ligands known as actives should be assembled. Second, the selection of decoys structures should depend on well-established criteria (e.g., DEKOIS 2.0 protocol [37,43,44]). Finally, to represent the ligand binding site well, a respective 3D structure is required. The eligible targets to produce benchmark sets are constrained by these fundamental requirements.

The main aim was to identify the best docking program that can distinguish between the active ligands and the created decoys efficiently. Accordingly, the effectiveness of the corresponding screening rises with an increasing active number recognized in the list of best-scored compounds.

Consequently, we created a challenging set of decoys by using the DEKOIS 2.0 protocol based on the collected bioactives of Fascin from the literature. Then, the benchmarking study was conducted using four popular docking tools, namely, MOE, AutoDock Vina, VinaXB, and PLANTS for evaluating their screening performance against the Fascin structure.

**Figure 2:**
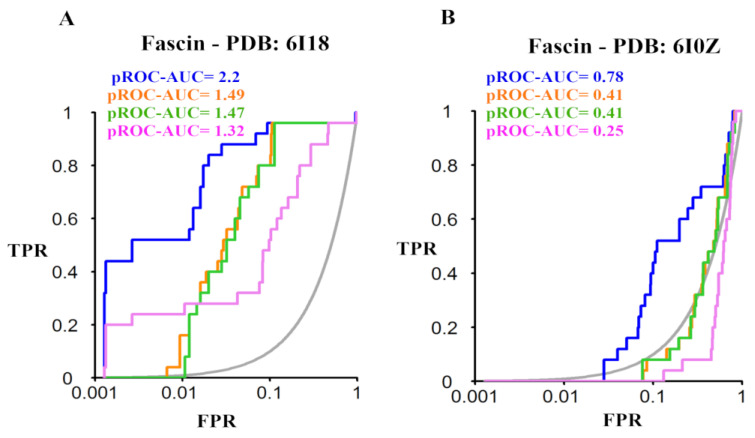
pROC plots of benchmarking analysis displaying the screening performance against Fascin (PDB ID: 6I18) and Fascin (PDB ID: 6I0Z) for (**A**,**B**), respectively. The curves of the docking tools PLANTS, Autodock vina, VinaXB, and MOE are represented by blue, orange, green, and magenta lines, respectively, while the grey line indicates the random screening performance. The true-positive rate (TPR), *y*-axis, represents the detected bioactives fraction, while the false-positive rate (FPR), *x*-axis, is the decoys retrieved fraction from a score − ordered list of all decoys.

The benchmarking results against Fascin (PDB ID: 6I18) and (PDB ID: 6I0Z) revealed that the four docking tools exhibited significantly better performance for Fascin (PDB ID: 6I18), compared to Fascin (PDB ID: 6I0Z). Furthermore, all assessed tools showed better-than-random performance against Fascin (PDB ID: 6I18), as shown in Figure 2A. Interestingly, PLANTS demonstrated the best screening performance in both structures indicated by a pROC-AUC (receiver operating characteristic-area under the curve) value of 2.2 for PDB: 6I18, compared to the pROC-AUC of 1.32, 1.49, and 1.47 for MOE, AutoDock Vina and VinaXB, respectively (Figure 2A). However, the crystal structure of Fascin (PDB ID: 6I0Z) yielded pROC-AUC values of 0.78, 0.25, 0.41, and 0.41 for PLANTS, MOE, AutoDock Vina, and VinaXB, respectively, as shown in (Figure 2B).

**Figure 3:**
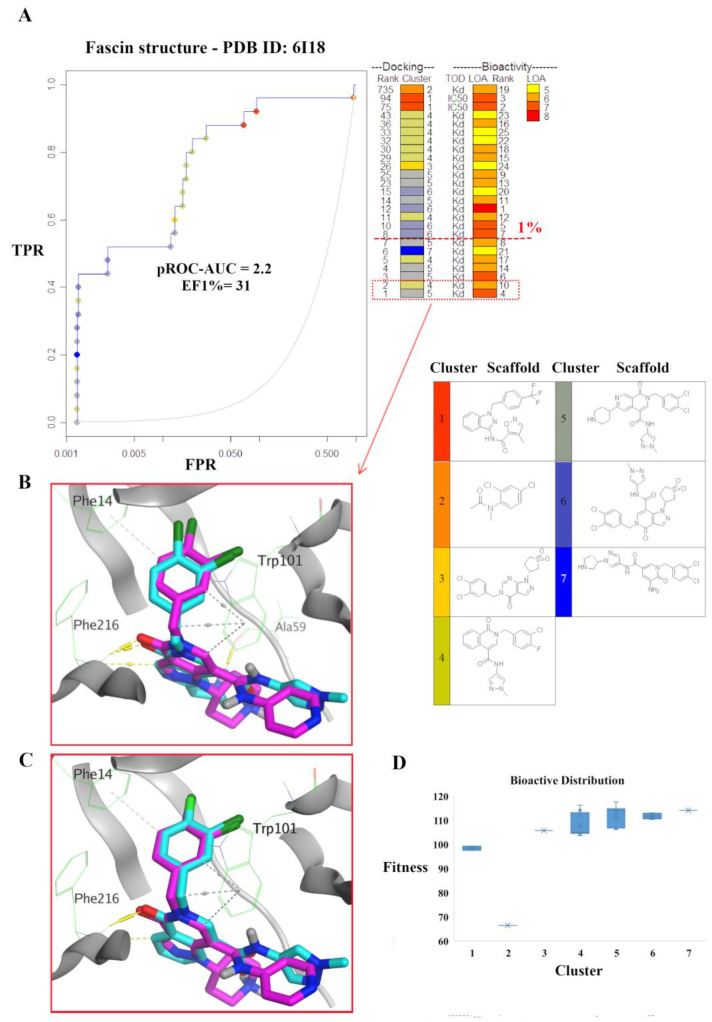
(**A**) pROC-Chemotype plot of the Fascin PDB ID: 6I18 using the PLANTS docking program. The docking data is coordinated with the cluster number and the bioactivity rank. The color scale of the bioactivity rank is from yellow (less potent) to red (more potent). A 1% bioactive enrichment is represented by the red-dashed line. (**B**,**C**) Docking poses of the best two ranked compounds overlaid on the co-crystal ligand (rank 12) as cyan and magenta sticks, respectively. (**D**) Bioactive molecules distribution is represented by a box plot of the fitness values *vs.* chemotype clusters.

The chemotype enrichment was analyzed with a “p-ROC-chemotype” [45,46] plot (Figure 3) for the benchmarking of the Fascin structure (PDB ID: 6I18) using a PLANTS docking program. We obtained different chemotype classes (7 clusters) based on their scaffolds. Generally, such clusters’ numbers reflect the lack of known small-molecule ligands that emphasizes the need of discovering more various small molecules working as Fascin inhibitors. The bioactivity data of the actives are represented by level of activity (LOA) values ranging from 10^5^ to 10^−8^ M and recorded as IC_50_ or Kd as a type of data (TOD) (Figure 3A).

The pROC-Chemotype plot revealed that PLANTS can detect potent binder ligands at early enrichment, as shown in (Figure 3A). For example, the two best-ranked molecules (with docking ranks 1 and 2) have bioactivity ranks 4 and 10, and Kd values of 250 and 1200 nM, respectively, Figure 3A. Both molecules exhibited interactions with the following residues; Phe216, Trp101, and Ala59 for compound rank 1, in addition to interactions with Phe216 and Phe14 for compound rank 2 (Figure S3) reproducing the key interactions of the reported ligand of Fascin (PDB ID: 6I18) as shown in (Figure 3B,C). It is noteworthy that this co-crystal ligand (BDP-13176) is involved in the active set with a docking rank of 12, and bioactivity rank of 1.

Moreover, only active molecules were recognized at 1% of the score-ordered molecules list, and none of the decoys were enriched, yielding an Enrichment Factor (EF 1%) of 31 for PLANTS compared to 23.31, 3.88, and 3.88 for MOE, Vina, and VinaXB, respectively. This indicates the potential predictive capability of PLANTS to identify active compounds 31 times at early enrichment (database cutoff 1%) more frequently than random performance.

Figure 3D displays the docking fitness distribution (fitness = docking score multiplied by −1) of the active compounds. The range of docking score started from −117.6 (best score) to −66.42 (worst score), reported as fitness values of 117.6 to 66.42. In addition, the compounds of cluster 5 are in the superior region of fitness values (e.g., fitness < 106).

Unlike the Fascin (PDB ID: 6I18), the screening performance of Fascin (PDB ID: 6I0Z) using PLANTS docking did not enrich any active compound at 1% of the database as shown in Figure 2B and its pROC-Chemotype plot (Figure S4). This observation is consistent with other assessed docking tools emphasizing the target-dependent nature of the benchmarking process. These results highlight that the binding site conformation of PDB: 6I18 is well-adapted for recognizing small molecule inhibitors and well-suited for virtual screening efforts. Therefore, the results encouraged us to apply PLANTS in the prospective VS against the Fascin structure (PDB ID: 6I18).

### 2.4. Prospective Virtual Screening

According to the promising benchmarking analysis results, we employed PLANTS in the virtual screening process of the FDA-approved drugs from the DrugBank database (1469 compounds) [47] besides the natural products from the Northern African Natural Products Database (3912 compounds) [48] against the Fascin structure (PDB ID: 6I18). The VS results of the best enriched 1% of the FDA-approved drugs and NANPDB are displayed in Table 2 and (Table S2), respectively. Regarding the molecules’ binding poses, they all showed comparable orientations and interactions with the key amino acids of the binding site 2 to the co-crystal ligand of PDB ID: 6I18, as seen in Figure 1. We chose to elucidate the binding interactions of the best ranked molecules which exhibited better localization of the Fascin binding site 2.

Visualizing the DrugBank results, they showed that Remdesivir, Lapatinib, and Fexofenadine, respectively appeared to be the top-scored compounds meeting the previous criteria.

**Figure 4:**
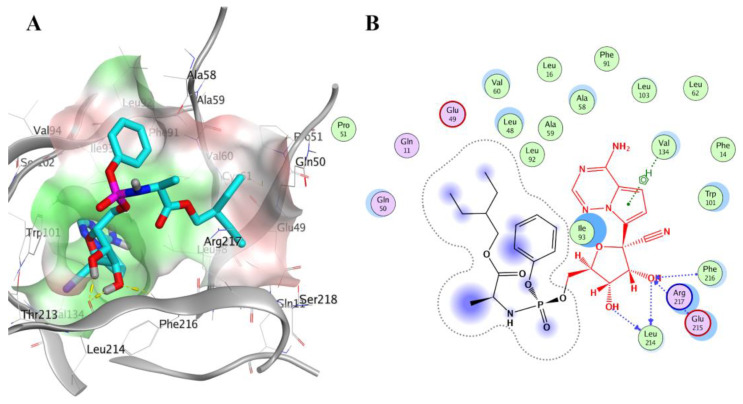
Docking pose of Remdesivir (DrugBank cyan sticks) in the Fascin binding site 2 (PDB ID: 6I18), represented by 3D and 2D as (**A**,**B**), respectively. Polar and non-polar areas of the pocket are shown in red and green colored molecular surfaces, respectively. Yellow dashed lines show the ligand interactions. Non-polar hydrogen atoms are ignored for clarification.

Figure 4A displays the docking pose of Remdesivir in the Fascin pocket (PDB ID: 6I18). Remdesivir is a nucleoside analog inhibiting RNA-dependent RNA polymerase. It is used to treat viral infections such as severe acute respiratory syndrome coronavirus 2 [49]. Its proposed binding pose in the Fascin pocket displayed H-bonding interactions via its dihydroxy groups with the side chains of Leu214 and Phe216, Figure 4B. We used the chemical structure of Remdesivir without further bioactivation.

Lapatinib is a tyrosine kinase inhibitor working as an antineoplastic agent that is used for treating patients with aggressive or metastatic HER-negative breast cancer who treated with previous chemotherapies [50]. Its postulated docking pose (Figure S5) made H-bond interactions through its methylsulfonyl group with Arg217 and Val248 side chains as well as H-pi interaction via its 3-flurophenyl group with Phe14.

Fexofenadine is a second-generation antihistamine that is considered a selective H1-receptor antagonist indicated for chronic idiopathic urticarial and allergic rhinitis treatment [51]. The binding pose of Fexofenadine exhibited H-bond interactions with Leu214 and Phe216 side chains, besides the H-pi interaction with Gln50 (Figure S6).

**Figure 5:**
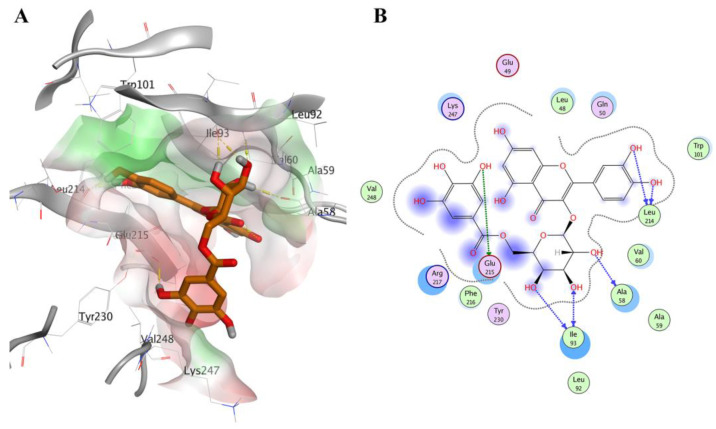
Docking pose of compound CP3756 (NANPDB3—orange sticks) in the Fascin binding site 2 (PDB ID: 6I18), represented by 3D and 2D as (**A**,**B**), respectively. The color scheme is the same as in Figure 4.

Regarding the VS of the natural products from NANPDB, compounds CP3451, CP3270, and CP3685 (see Table S2), termed NANPDB1-3 thereafter, displayed the best docking pose and ligands’ interactions occupying the binding site 2 of Fascin protein, effectively. Compound NANPDB1 is 2S,3R-4E,8E-2-(octadecanoylamino)-octadeca-4,8- diene-1,3-diol. It is ceramide that was extracted and isolated from the Egyptian Red Sea soft coral *Heteroxenia ghardaqensis*. The extracted compound exhibited a moderate anti-cancer effect on human Hep-G2 cancer cell lines working as a growth inhibitor [52]. NANPDB2 is 1-O-linoleoyl-3-O-beta-D-galactopyranosyl-syn-glycerol isolated from the aerial parts extract of the Egyptian plant *Sida spinosa* L., Malvaceae. The plant was reported to be used in treating nervous, urinary, and cardiac diseases [53]. NANPDB3 is Quercetin-3-O-beta-(6″-galloylgalactoside) which was isolated from the Egyptian *Sanguisorba minor* plant. The plant’s extract is used in folk medicine for its hypoglycaemic activity [54]. These natural products are expected to be used as adjuvant therapies or as supplements with cancer therapies. The docking poses and ligands’ interactions of the best-scored compounds CP3451 (NANPDB1), CP3270 (NANPDB2), CP3756, CP3407, and CP3831 are displayed in Figures S7–S11, respectively. NANPDB1 exhibited H-bond interactions with Ile93 while NANPDB2 displayed H-bond interactions with Glu215, Arg217, and Val248. The proposed binding pose of NANPDB3 made H-bond interactions with Glu215, Ala58, Ile93, and Leu214, as shown in Figure 5.

### 2.5. Molecular Dynamics Simulation

**Figure 6:**
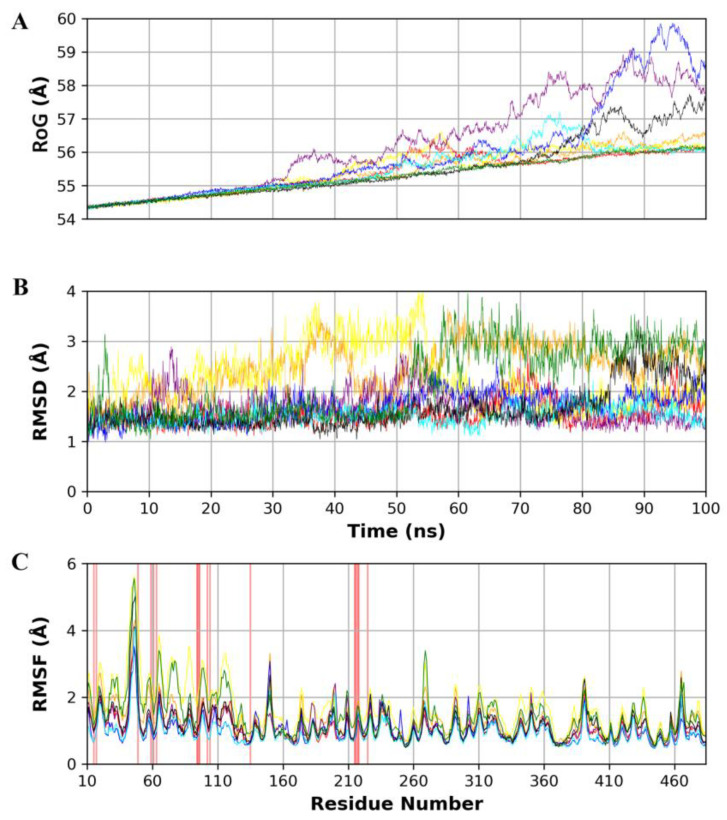
(**A**) Radius of gyration (RoG) of the protein throughout the simulation time. (**B**) Root mean square deviation (RMSD) of the protein alpha carbons throughout the simulation. (**C**) Per residue root mean square fluctuation (RMSF) with the amino acid residues of the binding site are presented with a red background. The color scheme is represented as the following: (red: Remdesivir, orange: Lapatinib, yellow: Fexofenadine, purple: NANPDB1, blue: NANPDB2, cyan: NANPDB3, black: Holoprotein, and green: unliganded protein).

The three top-enriched ligands in DrugBank (Remdesivir, Lapatinib, and Fexofenadine) and compounds (NANPDB1-3) from the natural products database were subjected to 100 ns MD simulations to evaluate their stability inside the Fascin binding site. Seven MD runs were conducted, including two extra runs for the holoprotein and unliganded protein as a reference to account for the dynamics of the protein and its co-crystallized ligand. Figure 6 displays the analysis of the protein’s radius of gyration (RoG), root mean square deviation (RMSD), and root mean square fluctuation (RMSF). Radius of gyration is a measurement of how compact the protein structure was throughout the simulation period. The RoG fluctuation of the protein complexes is within 6 Å, with Remdesivir and NANPDB2 having the lowest and the highest RoG values, respectively, at the end of the 100 ns simulation as shown in Figure 6. For instance, the reference holoprotein exhibited minor fluctuations until 75 ns, then a deviation can be observed afterward from 75 ns to the end of the simulation within 56 Å to 58 Å. While the unliganded system displayed a smooth increase of RoG from 54 Å to 56 Å with no visible dramatic fluctuations on its path. Unlike the behavior of RoG of the reference simulations, NANPDB1 (purple) and NANPDB2 (blue) showed obvious high fluctuations after 30 ns and 80 ns of simulation time, respectively, compared to other ligand-complex systems. Nonetheless, the overall RoG behavior of all complexes indicates successful protein simulation during the simulation course and the absence of major conformational changes or unfolding processes during the simulation.

Moreover, the protein dynamics’ stability was assessed via the RMSD, which was calculated on alpha carbon atoms. For the unliganded protein, low change for the RMSD values can be observed until 50 ns of simulation; afterward, a higher change took place from 1.5 Å to 3 Å, while the holoprotein complex displayed a lower change throughout the simulation time with low fluctuation after 85 ns. This suggests that the co-crystal ligand can better stabilize the protein and produce lower fluctuations for the backbone protein atoms. Interestingly, Remdesivir (red), NANPDB1 (purple), NANPDB2 (blue), and NANPDB3 (cyan) displayed lower changes in the RMSD values compared to Lapatinib (orange) and Fexofenadine (yellow). For instance, the Remdesivir complex system displayed RMSD values around 1.5 Å from 0 to 65 ns, then rising to 2.5 Å at 70 ns to return around 1.5 Å from 75 to 95 ns, with another cycle of rising to 2.5 Å at 95 ns and coming back to 1.5 Å at 100 ns. Unlike the low RMSD value fluctuations of Remdesivir, Fexofenadine showed increased values from 2 Å to 4 Å from 0 to 55 ns simulations, while rapid increase and fluctuations could be seen around 2 Å from 55 to 100 ns. Initially, these observations indicate the superior ability of Remdesivir from the FDA-approved drugs to stabilize protein backbone compared to Lapatinib and Fexofenadine, and likewise, for the three natural products.

RMSF measures the per residue conformational changes throughout the simulation time. We observed common flexibility patterns in all systems in some regions, mainly in residue numbers 30–50 with RMSF values (>3 Å) and 130–150 (>2 Å). This observed flexibility is attributable to the structural loop regions for the Fascin protein. The flexibility of these regions was significantly reduced in systems with Remdesivir (red), NANPDB2 (blue), and NANPDB3 (cyan), especially compared to the unliganded system (green) and the co-crystal system (black). For instance, the region of residue number 30–50 exhibited >5 Å fluctuations for both the unliganded and co-crystal systems, while the presence of Remdesivir (red), NANPDB2 (blue), and NANPDB3 (cyan) as ligands reduced such fluctuations dramatically (<4 Å). On the other hand, other systems, such as Fexofenadine (yellow) displayed high flexibility in the majority of the regions. Remarkably, complex systems with Remdesivir (red), NANPDB2 (blue), and NANPDB3 (cyan) exhibited low RMSF values (<2 Å) at the binding site amino acids revealing the minimal conformational changes for these residues and reflecting a promising stabilization effect of these ligands to the binding site.

**Figure 7:**
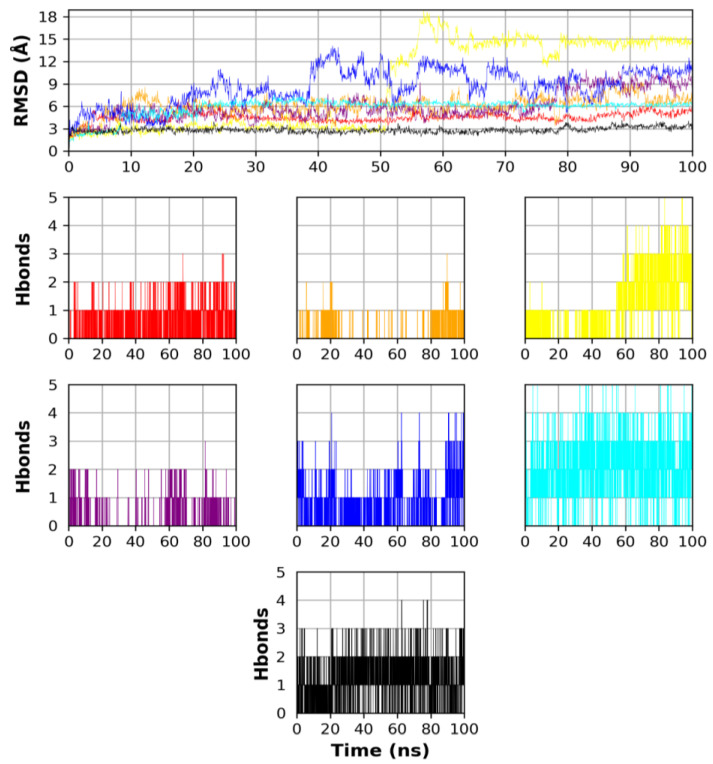
Ligand RMSD and hydrogen bond analysis. The graphs of Remdesivir, Lapatinib, and Fexofenadine are shown in red, orange, and yellow, respectively, while the graphs of NANPDB1, NANPDB2, NANPDB3, and Holoprotein are shown in purple, blue, cyan, and black, respectively.

Figure 7 shows the analysis of RMSD for the heavy atoms of the ligand poses and their hydrogen bond count with the protein. As a reference, the co-crystal ligand showed stable RMSD behavior around 3 Å throughout the whole simulation reflecting good stability of the co-crystal pose. According to RMSD measurements for the tested poses, Remdesivir (red) displayed the lowest RMSD values among the other poses of the FDA-approved drugs revealing its highest stability within the Fascin binding site. Remdesivir showed minor fluctuations from 3 Å to 5 Å at 0 ns to 5 ns, then a steady behavior around 5 Å ± 1 Å from 5 ns to 100 ns. On the other hand, Fexofenadine (yellow) exhibited a balanced behavior around 3 Å from the start of the simulation to 50 ns, then a rapid increase in RMSD values to 12–18 Å after 50 ns until the end of the simulation, reflecting high changes in the pose coordinates in the binding site. From the natural product ligands, NANPDB3 (cyan) showed the best stability compared to NANPDB1 (purple) and NANPDB2 (blue). The NANPDB3 pose exhibited an increase in RMSD values from 3 Å to 6 Å during the 0 ns to 20 ns, while it showed a constant performance around the RMSD value of 6 Å afterward throughout the simulation from 20 ns to 100 ns. Unlike NANPDB3, NANPDB2 displayed high RMSD fluctuations, especially after 20 ns of the simulation time where the RMSD values increased towards 14 Å at 39–43 ns with some greater fluctuations until the end of the 100 ns simulation.

According to the generated hydrogen bonds number between each ligand and its relevant protein (Figure 7), NANPDB3 (cyan) exhibited the highest number of hydrogen bonds revealing its potent binding compared to other molecules, followed by NANPDB2 (blue) and Fexofenadine (yellow) showing their moderately strong affinity. Regarding Lapatinib and NANPDB1, they exhibited the least number of hydrogen bonds formed with their proteins indicating their weak affinity. Although Fexofenadine showed a high number of hydrogen bonds at the end of the simulation, we propose that its pose underwent a major positional change inside the binding site provided by the fact of its RMSD plot (Figure 7).

**Figure 8:**
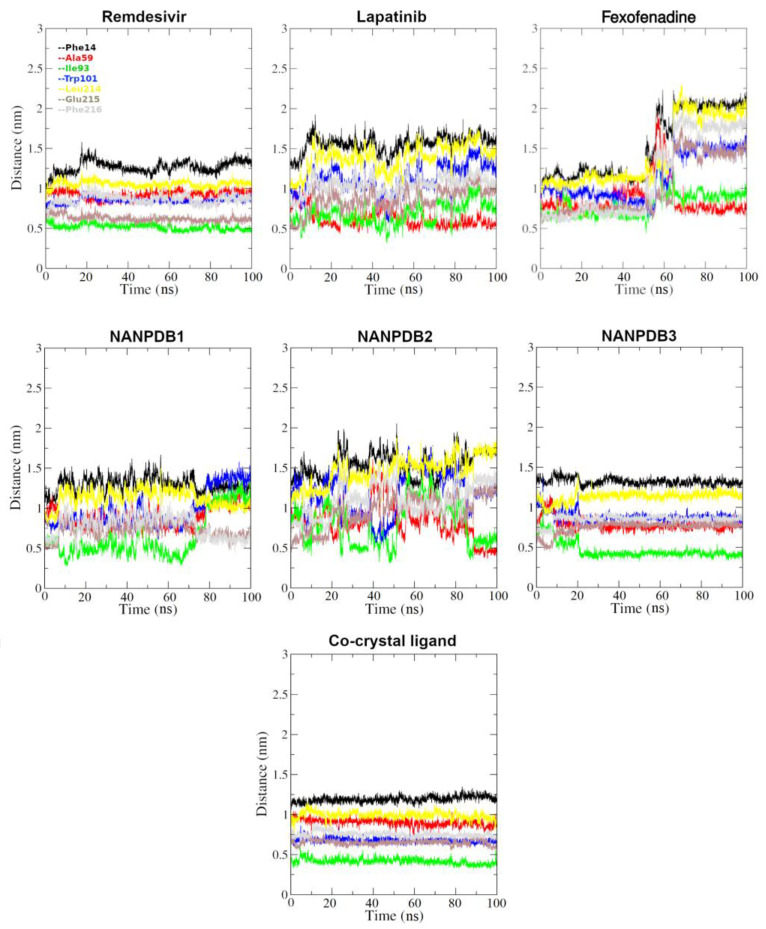
The distance between the center of mass of the indicated ligand and residues of Fascin protein in the binding site under investigation during a 100 ns (100,000 ps) MD simulation.

To further elucidate the relative positioning of the proposed ligands to the key binding site residues, we monitored their relative distances (based on the center of mass) to such residues of the Fascin binding site during the 100 ns simulation course. The selection of residues to be considered in this assessment was based on their role in the ligand’s binding to the Fascin binding site 2. The reported Fascin crystal structures revealed that the ligands bind in an induced pocket with a hydrophobic “hook” in the cleft between the β-trefoil domains 1 and 2 and extend towards the protein surface, causing a significant conformational change in domain 1 and making interactions with specific domain 2 residues [24,27]. Although the inhibition mechanism of the Fascin bundling activity is still unclear, it is noteworthy that proposed Fascin’s actin-binding sites 1 and 2 include domain 1 crossing a domain boundary. Moreover, both sites would be deformed by the conformational change induced by the bound ligand, thus disrupting actin-binding [24,27]. Accordingly, Phe14, Ala59, Ile93, and Trp101 residues were selected from domain 1 besides Leu214, Glu215, and Phe216 from domain 2 for the distance monitoring to the proposed ligands. Visualizing DrugBank candidates, Remdesivir exhibited the best behavior compared to Lapatinib and Fexofenadine indicated by its distance behavior to the selected residues, as observed in Figure 8. For instance, Remdesivir displayed a distance range of ~(1–1.6), (0.9–1), (0.5–0.6), 0.75, (0.9–1.1), (0.6–0.7), and (0.6–1) nm to the center of mass of Phe14, Ala59, Ile93, Trp101, Leu214, Glu215, and Phe216, respectively. Such behavior with minor distance range fluctuations, especially after 20 ns, best mimicked the behavior of the co-crystallized ligand (Figure 8), reflecting an acceptable stability of Remdesivir during the simulation course. However, the distance values of Lapatinib and Fexofenadine indicated higher fluctuations per residue compared to the co-crystallized ligand, as seen in Figure 8. Like Remdesivir, NANPDB3 displayed the best distance behavior compared to NANPDB1 and NANPDB2, especially after 20 ns. NANPDB3 displayed distance ranges of ~(1.25–1.5), (0.75–1.1), (0.75–0.3), (0.8–1.25), (0.9–1.1), (0.5–0.75), and (0.6–0.8) nm to the center of mass of Phe14, Ala59, Ile93, Trp101, Leu214, Glu215, and Phe216, respectively. Again, such distance behavior highlights low fluctuations and satisfactory stability of NANPDB3 in the binding site, in a comparable manner to the co-crystallized ligand.

Based on the above-mentioned results, both Remdesivir (from DrugBank) and NANPDB3 (from NANPDB) showed high stability and were recommended to be the best potential binders to Fascin actin-binding site 2.

**Figure 9:**
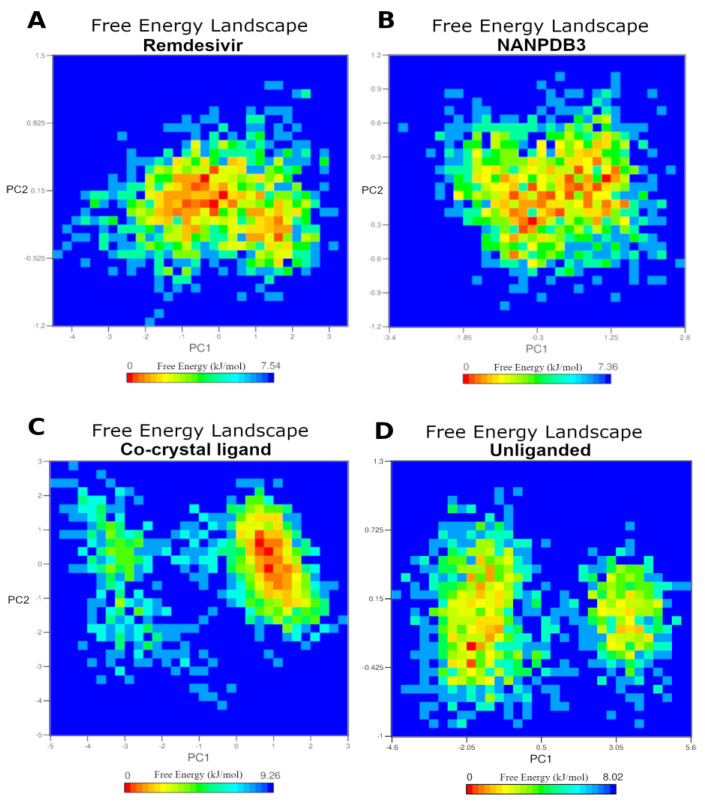
The free energy landscape (FEL) of the simulated Fascin systems based on the principal component analysis. (**A**) Remdesivir − Fascin complex system. (**B**) NANPDB3 − Fascin complex system. (**C**) The co − crystal ligand − Fascin complex system. (**D**) The unliganded − Fascin system. The color bar represents the free energy value in kcal mol ^−1^. The color ranges from red to yellow to blue spots indicate the energy minima and energetically favored protein conformations to more unfavorable high-energy conformations.

Focusing on Remdesivir and NANPDB3, we utilized the principal component analysis (PCA) to analyze the conformational sampling of the Remdesivir-, NANPDB3- and co-crystallized ligand-Fascin complex systems, as well as the unliganded protein system in the simulated subspace via examining their dominant modes of motion. The covariance matrix of atomic fluctuations was diagonalized for predicting the eigen values. The first few eigen vectors play a critical role in the motions of the protein. The first 2 eigen vectors have a higher eigen value for all four Fascin systems suggesting—to a certain extent—their comparable behavior for the whole protein motion. To expose the ligand influences on the conformational heterogeneity of Fascin, associated free energy landscapes (FEL) were determined as a function of the top two principal components (PC1 and PC2), as exemplified in Figure 9. FEL can be employed to effectively explain conformational redistributions prompted by binding events [55,56,57].

Figure 9 demonstrates the relative conformational changes of the protein backbone of the four simulated systems. The deeper color (towards the red color) in the plot reveals lower-energy conformational metastable states. Remdesivir- and NANPDB3-Fascin complex systems populated by a wide energy basin suggesting a range of metastable states and ensemble of low energy conformations of the simulated subspace during the 100 ns simulation (Figure 9A,B). On the other hand, the simulated co-crystal system of Fascin visits two separate energy basins; one represents the global minimum of the simulated subspace, while the other is quite narrow and separated by some conformations with relatively low energy from the main basin (Figure 9C). This reflects the presence of one main ensemble of low energy conformations of flexible and low energy conformations during 100 ns simulation. Remarkably, the unliganded protein system (Figure 9D) clearly displays two distinct energy basins with low incidence for visiting the global minimum of the simulated subspace (few red dots). This reflects that the liganded complex systems (Figure 9A–C) would drive the simulated subspace into a higher incidence of lower energy ensemble of conformations compared to the unliganded system. Thus, these results clearly highlight that Remdesivir and NANPDB3 binding to Fascin can alter the protein conformational subspace towards low-energy conformations, and therefore, modulate its function.

Overall, the results of the MD simulations endorse the high potential and stable binding of Remdesivir and NANPDB3 to Fascin as an outcome of a benchmarking-guided virtual screening effort.

**Figure 10:**
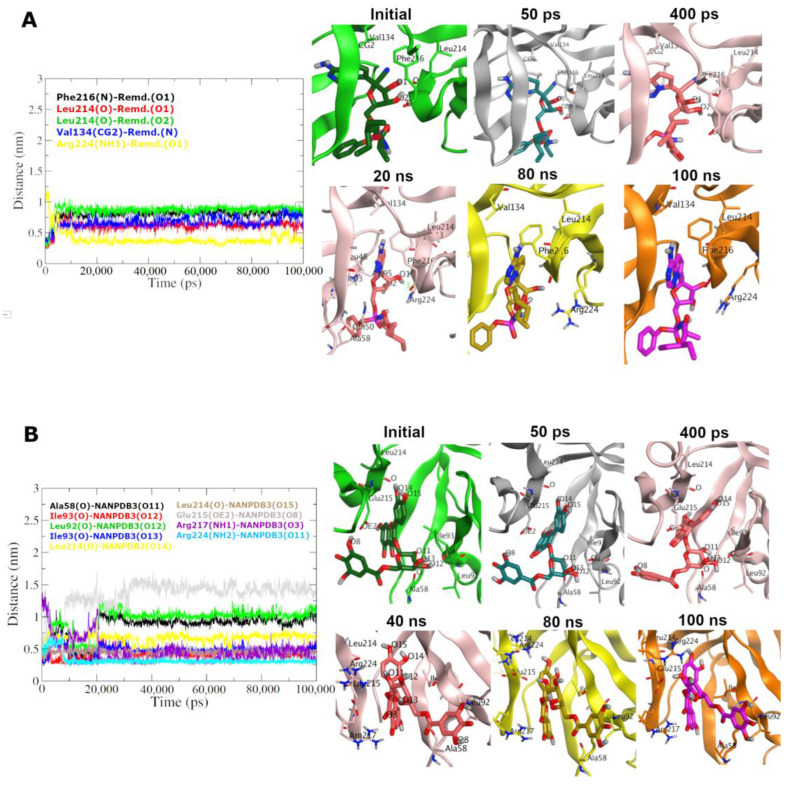
The minimum distance graph of both Remdesivir- and NANPDB3- Fascin interactions for (**A**,**B**), respectively, during the MD simulation. Snapshots at different simulation time are illustrated for both systems.

To provide more insights on both poses of Remdesivir and NANPDB3 throughout the MD trajectory, we computed the minimum distance between the interacting atoms of the ligand and protein residues, as shown in Figure 10. Initially, Remdesivir showed H-bonding interactions with Leu214 and Phe216, as well as hydrophobic interaction with Val134. These favorable interactions are reflected in distances of ~0.3 to 0.4 nm (i.e., 3 Å – 4 Å) between Remdesivir’s O1 and O2 atoms of its sugar-like moiety to the O atoms of the backbone of Leu214 and Phe216 (more details are in Figure 10). This interacting pose appeared to be consistent during the beginning of the simulation time (from 0 to 5 ns), while the distance graph proposes that some dynamics affected the interaction pattern with a new interacting pose at 10 ns and remained consistent throughout the whole 100 ns MD simulation. During this transformation, a new interacting residue (Arg224 – yellow line in Figure 10A) approached to form H-bonding interactions with Remdesivir (distance ~0.3 to 0.4 nm from atom O1) stabilizing its new pose from 10 ns to 100 ns of the simulation time. Interestingly, Leu214, Phe216 and Val134 remained at constant distance ranges from 0.5 nm to 0.8 nm. Inspecting the relative position of different poses of Remdesivir, we conclude that there are two main clusters of Remdesivir poses revealed via different time snapshots. The first cluster of poses can be visualized during simulation time of 0-5 ns, while the second cluster comprises most of the poses, during 5 ns to 100 ns of the simulation time (see the snapshots in Figure 10). The main differences between the two cluster of poses are attributable to minor rotation of the heterocyclic system of pyrrolotriazine ring and the bonded sugar-like part to accommodate favorable binding and H-bonding interactions in the binding site. These observations propose a stable binding of Remdesivir at the near proximity of the key Fascin residues and hence potentially modulate its function.

Like Remdesivir, NANPDB3 pose initially displayed H-bonding interactions with Leu214, and additionally with Glu215, Ile93 and Ala58. The key atoms for this H-bonding network are the O atoms for the sugar part, namely: O11, O12 and O13 and the O atoms of the backbone of the mentioned residues and the side chain oxygen (OE2) of Glu215 (see Figure 10B). Such favorable interactions are reflected in distances of ~0.3 to 0.4 nm between the respective atoms, as displayed in Figure 10B. The interacting NANPDB3 pose exhibited some fluctuations from 0 to 20 ns time with a distance range to the respective residue atoms from 0.3 to 1.75 nm (Figure 10B). A new stable pose is predominantly formed from 20 ns to the end of 100 ns simulation time creating new balanced distances to the side chain atoms of NH1 and NH2 for Arg217 and Arg224, respectively, indicating stable H-bonding interactions. Taking different time snapshots of NANPDB3 poses (Figure 10B) revealed that the new pose (time: 20–100 ns) is evolved due to a major flip of the trihydroxy phenyl group of NANPDB3 from its early poses (time: 50 ps and 400 ps). This flip is tolerable since the trihydroxy phenyl group is mostly solvent-exposed and possess greater degrees of freedom. Overall, like Remdesivir, these observations suggest a stable binding of NANPDB3 after 20 ns at the vicinity of the key Fascin residues and therefore hypothetically able to modulate its function. Generally, these observations of Figure 10 are highly consistent with the observations of Figure 8.

## 3. Materials and Methods

### 3.1. Preparation of Protein Structures

Crystal structures (PDB ID. 6I0Z) and (PDB ID. 6I18) of Fascin, adopted for this study, were isolated from homo sapiens and expressed in *Escherichia coli*. The structures are co-crystallized in complex with ~{N}-(2,4-dichlorophenyl)-~{N}-methyl- ethanamide and 5-[(3,4-dichlorophenyl)methyl]-4-oxidanylidene-1-piperidin-4-yl-~{N}-pyridin-4-yl- pyrazolo [4,3-c]pyridine-7-carboxamide, respectively. The X-ray crystal structures were downloaded from the Protein Data Bank where they displayed Resolutions of 1.77 Å and 1.49 Å, respectively.

Molecular Operating Environment (MOE) v.2019.01 [58] was utilized to prepare the protein structures before the docking processes, adopting the AMBER10:EHT force field. Any redundant chains with unessential ions, crystallization molecules, and water molecules were discarded. Then, the MOE function “Quickprep” was employed at default settings. These settings include applying the function “Protonate 3D” for improving the H-bonding network and permitting ASN/GLN/HIS to flip throughout protonation. In addition, the refinement of the ligand and binding site atoms was conducted by minimizing the energy to 0.1 kcal/mol/A RMS gradient, and the receptor atoms were restrained by applying a force constant (strength = 10). The remaining atoms of the receptor lying outside the binding pocket were maintained the same. The outcome of the previous settings did not display a significant difference regarding the binding site/ligand coordinates. The prepared protein structures were saved as mol2 files for the docking efforts. The benchmarking experiments were conducted on Fascin (PDB ID: 6I0Z) and Fascin (PDB ID: 6I18), while Fascin (PDB ID: 6I18) was chosen for VS of the DrugBank and NANPDB molecules.

MOE v.2019.01 was used for conducting the protein superpositions.

### 3.2. Preparation of Small Molecules of DEKOIS 2.0 Benchmark Set, DrugBank FDA-Approved Drugs, and NANPDB Molecules

The DEKOIS 2.0 [37] protocol was employed on 25 Fascin bioactives, collected from literature [23,24,27], to create 750 challenging decoys (1:30 ratio). After that, preparation of all molecules was performed by MOE v.2019.01 using ‘Molecule wash’. This setting was utilized to produce valid protonation states through protonating strong bases and deprotonating strong acids (if needed). In addition, the minimizing of compounds energy was employed via forcefield “Amber: 10EHT” at a 0.01 RMSD gradient. The remaining parameters were maintained at default settings. One protonation state was made at pH 7.0 and one conformer was retained for each molecule. Moreover, the stereo configuration of all actives, decoys, DrugBank, and NANPDB compounds was kept. The prepared compounds were saved as SD files which were transformed and split into PDBQT files via OpenBabel [59] for AutoDock Vina and VinaXB docking experiments. For docking experiments via PLANTS, the SD files were converted into mol2 files and the types of correct atoms were performed by SPORES software [60,61].

### 3.3. Docking Experiments

#### 3.3.1. Benchmarking

Concerning docking of the prepared molecules to the active site of Fascin structures using MOE v.2019.01, the molecules were docked in the ligand binding site of the Fascin structure. Triangle matcher was set as the placement, while London dG and GBVI/WSA dG were set as the first and second rescoring functions, respectively and the refinement was via forcefield.

Regarding docking via AutoDock Vina (version 1.1.2) and VinaXB [40,42], the converting of the protein files to PDBQT files was performed by utilizing a python script known as (prepare_receptor4.py) from the MGLTools package (version 1.5.4) [62]. The dimensions of the docking grid box were 18 Å × 18 Å × 18 Å, with a 1 Å grid spacing to ensure that all docked compound geometries were covered. However, the docking method’s search efficiency was retained at its default setting.

For PLANT (version 1.2) docking [41], “ChemPLP,” was the employed scoring function with selecting “screen” mode. Within 5 Å of the co-crystal ligand coordinates, the binding site was identified.

#### 3.3.2. Virtual Screening of DrugBank FDA-Approved Drugs and NANPDB Molecules

PLANTS was chosen as the docking program for virtual screening due to its significant performance in the benchmarking study. VS was carried out by docking FDA-approved drugs from DrugBank and NANPDB molecules into the prepared Fascin crystal structure in complex with the co-crystallized ligand BDP-13176 (PDB ID: 6I18).

### 3.4. pROC and pROC-Chemotype Calculations

The score-based docking rank was employed in the calculation of pROC-AUC utilizing the KNIME “R-Snippet” component [63] according to the following Equation (1) [64]:

(1) $$pROC\;AUC=\frac{1}{n}\;\overset{n}{\underset{i}{\sum }}[-\log_{10}\left(D_{i}\right)]=\frac{1}{n}\;\overset{n}{\underset{i}{\sum }}\log_{10}\left(\frac{1}{D_{i}}\right)$$

where *n* is the bioactives number while the decoys fraction ordered higher than *ith* active identified is represented by *Di* where *ith* is the bioactive number in the rank, and where *ith* represents the bioactive number in the rank.

The plots of pROC-Chemotype were generated by the tool “pROC-Chemotype plot” which is available at http://www.dekois.com/ (accessed on 15 July 2022) [45,46].

To evaluate the docking program’s ability to detect true-positive actives, in the list of the docking rank in comparison to the random collection, the enrichment factor (EF) was calculated according to the subsequent Equation (2) [65]:

(2) $$EF=\frac{Bioactives_{subset}}{N_{subset}}/\frac{Bioactives_{total}}{N_{total}}$$

The figures of protein structure were rendered using MOEv.2019.01 and Pymol [66].

### 3.5. Molecular Dynamics Simulations

Molecular dynamics simulations were conducted using GROMACS 2020.3 [67]. The solvation of each protein-ligand complex was carried out in a dodecahedron box of TIP3P explicit water model [68]. Then, the system was neutralized using NaCl ions with ionic strength of 0.1 M concentration. For system energy minimization, the steepest descent minimization algorithm was utilized by a convergence set at 10 kJ/mol and 50,000 steps. At 300 K temperature and 1 atm pressure, each NVT followed by NPT equilibration was conducted for 500 ps. After that, a production run at NPT ensemble was performed for 100 ns. For each equilibration run, temperature coupling was carried out using the V-rescale modified Berendsen thermostat [69], For equilibration and production runs, a 2 ps time constant Berendsen coupling [70] was employed for pressure coupling. Furthermore, for pressure coupling, the Parrinello-Rahman pressure coupling scheme [71] was utilized for the production runs. Using the Verlet cutoff-scheme with 1.2 cutoff and 1.0 nm switch list distances was for Van der Waals calculations and searching for adjacent atoms. The method of Particle Mesh Ewald [72] was employed for the long-range electrostatics calculations within 1.2 nm. The bond lengths were constrained using the LINear Constraint Solver (LINCS) algorithm. [73]. The protein molecules’ topology and parameters were generated by applying the CHARMM36 all-atom force field [74], while the ligand parameters were generated using the SwissParam server [75]. A leap-frog integrator with a steps size of 2 fs was utilized for all simulations. ProDy’s Python library was used to calculate protein RMSD, RMSF, and radius of gyration [76,77], while VMD’s rmsd trajectory analysis tool was used to determine ligand RMSD and hydrogen bonds [78]. GROMACS and Matplotlib python plotting library were employed for constructing all analysis charts [79].

## 4. Conclusions

Fascin is overexpressed in various carcinomas that are associated with metastasis and poor prognosis. In this study, we carried out (CADD) approaches to systematically recommend potential inhibitors of the Fascin protein. First, Fascin protein structures (PDB ID: 6I18) and (PDB ID: 6I0Z) were selected to represent the conformations of the target space of Fascin-liganded structures. Then, diverse bioactive molecules were collected from literature having different scaffolds, namely: Indazole, N-phenylacetamide, pyrazolo [3,4-d]pyrimidin-4-one, isoquinolone, naphthyridone, pyrazolo [4,3-c]pyridine and pyridone, to compile an active set for benchmarking study. Accordingly, a set of high-quality decoys was generated via DEKOIS 2.0 protocol to be utilized in the benchmarking process against the selected Fascin structures. Four popular docking tools, MOE, AutoDock Vina, VinaXB, and PLANTS were employed in the benchmarking effort. All docking tools exhibited better-than-random performance against one Fascin structure (PDB ID: 6I18). Based on the benchmarking outcomes utilizing the pROC-AUC, and EF 1%, PLANTS exhibited the best screening performance. Visualizing chemotype enrichment of PLANTS via a pROC-Chemotype plot revealed the ability of this docking tool to enrich the potent bioactive molecules in the early enrichment. This outcome encouraged us to employ PLANTS in conducting SBVS against Fascin (PDB ID: 6I18) to repurpose FDA-approved drugs (from DrugBank) and natural products (from NANPDB). The VS results showed that Remdesivir, Lapatinib, and Fexofenadine (from DrugBank) and NANPDB1-3 (from NANPDB) can be endorsed as potential binders of the Fascin structure. Finally, to further validate the compounds’ stability, we performed molecular dynamic (MD) simulations for 100 ns. MD recommended that Remdesivir from the DrugBank series and NANPDB3 from the NANPDB series to be the best potential binders to Fascin binding site 2.

Generally, our study provides an example of recruiting a DEKOIS 2.0 benchmark set as a method to elevate the success rate for further virtual screening efforts against new vital targets for anticancer and antimetastatic drug discovery.

In addition, the best-ranked repurposed molecules Remdesivir and NANPDB3 from FDA-approved drugs and natural products databases, respectively, are recommended for further biological investigations against Fascin to provide potential therapeutic agents.

## Figures and Tables

**Table 1:** The active set of Fascin inhibitors.

Scaffold/Cluster	Structure	Chemical Name	IC_50_ (µM)	Kd (µM)	Ref.
Indazole /Cluster 1	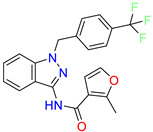	2-methyl-N-(1-(4-(trifluoromethyl)benzyl)-1H-indazol-3-yl)furan-3-carboxamide	0.2	nd	[23]
	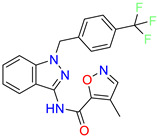	4-methyl-N-(1-(4-(trifluoromethyl)benzyl)-1H-indazol-3-yl)isoxazole-5-carboxamide	0.19	nd	[24]
*N*-Phenylacetamide /Cluster 2	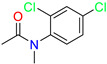	N-(2,4-dichlorophenyl)-N-methylacetamide	nd	92	[27]
Pyrazolo[3,4-d]pyrimidin-4-one /Cluster 3	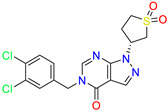	1-[(3~{R})-1,1-bis(oxidanylidene)thiolan-3-yl]-5-[(3,4-dichlorophenyl)methyl]pyrazolo [3,4-d]pyrimidin-4-one	67.6	29.5	
Isoquinolone /Cluster 4	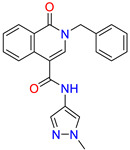	~{N}-(1-methylpyrazol-4-yl)-1-oxidanylidene-2-(phenylmethyl)isoquinoline-4-carboxamide	67.9	29.3	
	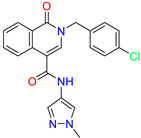	2-[(4-chlorophenyl)methyl]-~{N}-(1-methylpyrazol-4-yl)-1-oxidanylidene-isoquinoline-4-carboxamide	4.6	2.7	
	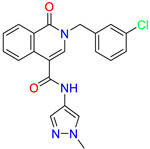	2-[(3-chlorophenyl)methyl]-~{N}-(1-methylpyrazol-4-yl)-1-oxidanylidene-isoquinoline-4-carboxamide	11.4	7.6	
	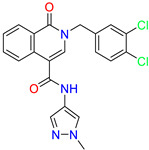	2-[(3,4-dichlorophenyl)methyl]-~{N}-(1-methylpyrazol-4-yl)-1-oxidanylidene-isoquinoline-4-carboxamide	1.3	1.5	
	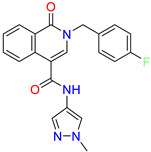	2-(4-fluorobenzyl)-N-(1-methyl-1H-pyrazol-4-yl)-1-oxo-1,2-dihydroisoquinoline-4-carboxamide	nd	2.7	
	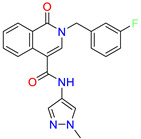	2-(3-fluorobenzyl)-N-(1-methyl-1H-pyrazol-4-yl)-1-oxo-1,2-dihydroisoquinoline-4-carboxamide	nd	29.2	
	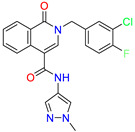	2-(3-chloro-4-fluorobenzyl)-N-(1-methyl-1H-pyrazol-4-yl)-1-oxo-1,2-dihydroisoquinoline-4-carboxamide	2.1	1.2	
	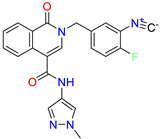	2-(4-fluoro-3-isocyanobenzyl)-N-(1-methyl-1H-pyrazol-4-yl)-1-oxo-1,2-dihydroisoquinoline-4-carboxamide	8.6	6.6	
	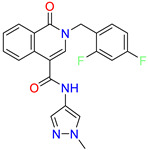	2-(2,4-difluorobenzyl)-N-(1-methyl-1H-pyrazol-4-yl)-1-oxo-1,2-dihydroisoquinoline-4-carboxamide	nd	46	
Naphthyridone /Cluster 5	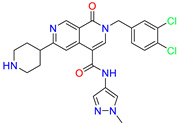	2-[(3,4-dichlorophenyl)methyl]-~{N}-(1-methylpyrazol-4-yl)-1-oxidanylidene-6-piperidin-4-yl-2,7-naphthyridine-4-carboxamide	0.51	0.25	
	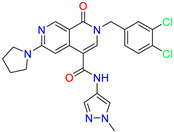	2-(3,4-dichlorobenzyl)-N-(1-methyl-1H-pyrazol-4-yl)-1-oxo-6-(pyrrolidin-1-yl)-1,2-dihydro-2,7-naphthyridine-4-carboxamide	nd	1.03	
	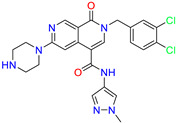	2-(3,4-dichlorobenzyl)-N-(1-methyl-1H-pyrazol-4-yl)-1-oxo-6-(piperazin-1-yl)-1,2-dihydro-2,7-naphthyridine-4-carboxamide	0.63	0.58	
	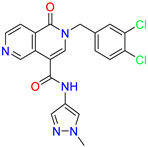	2-(3,4-dichlorobenzyl)-N-(1-methyl-1H-pyrazol-4-yl)-1-oxo-1,2-dihydro-2,6-naphthyridine-4-carboxamide	5.3	1.6	
	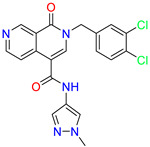	2-(3,4-dichlorobenzyl)-N-(1-methyl-1H-pyrazol-4-yl)-1-oxo-1,2-dihydro-2,7-naphthyridine-4-carboxamide	3.8	1.3	
	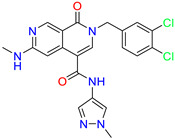	2-(3,4-dichlorobenzyl)-N-(1-methyl-1H-pyrazol-4-yl)-6-(methylamino)-1-oxo-1,2-dihydro-2,7-naphthyridine-4-carboxamide	nd	1.1	
	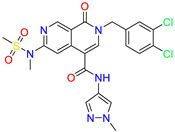	2-(3,4-dichlorobenzyl)-N-(1-methyl-1H-pyrazol-4-yl)-6-(N-methylmethylsulfonamido)-1-oxo-1,2-dihydro-2,7-naphthyridine-4-carboxamide	nd	2	
Pyrazolo[4,3-c]pyridine /Cluster 6	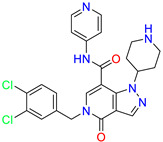	5-(3,4-dichlorobenzyl)-4-oxo-1-(piperidin-4-yl)-N-(pyridin-4-yl)-4,5-dihydro-1H-pyrazolo [4,3-c]pyridine-7-carboxamide	0.24	0.09	
	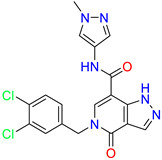	5-(3,4-dichlorobenzyl)-N-(1-methyl-1H-pyrazol-4-yl)-4-oxo-4,5-dihydro-1H-pyrazolo [4,3-c]pyridine-7-carboxamide	10.4	10	
	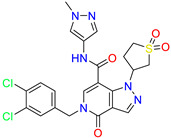	5-(3,4-dichlorobenzyl)-1-(1,1-dioxidotetrahydrothiophen-3-yl)-N-(1-methyl-1H-pyrazol-4-yl)-4-oxo-4,5-dihydro-1H-pyrazolo [4,3-c]pyridine-7-carboxamide	0.6	0.6	
	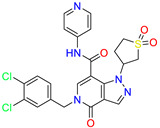	5-(3,4-dichlorobenzyl)-1-(1,1-dioxidotetrahydrothiophen-3-yl)-4-oxo-N-(pyridin-4-yl)-4,5-dihydro-1H-pyrazolo [4,3-c]pyridine-7-carboxamide	0.33	0.27	
Pyridone /Cluster 7	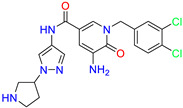	5-amino-1-(3,4-dichlorobenzyl)-6-oxo-N-(1-(pyrrolidin-3-yl)-1H-pyrazol-4-yl)-1,6-dihydropyridine-3-carboxamide	<100	21	

**Table 2:** The best enriched 1% of the VS results for FDA-approved drugs against Fascin (PDB ID: 6I18).

Docking Rank	Drug	Docking Score	M.wt.	Drugbank ID	Status
1	Remdesivir	−124.43	602.59	DB14761	Approved; investigational
2	Lapatinib	−119.231	581.06	DB01259	Approved; investigational
3	Fexofenadine	−119.101	501.66	DB00950	Approved; investigational
4	Latanoprost	−118.59	432.59	DB00654	Approved; investigational
5	Almitrine	−118.311	477.55	DB01430	Approved
6	Fulvestrant	−116.406	606.78	DB00947	Approved; investigational
7	Travoprost	−116.314	500.55	DB00287	Approved
8	Indinavir	−115.639	613.79	DB00224	Approved
9	Vilazodone	−114.94	441.52	DB06684	Approved
10	Oxetacaine	−114.377	467.65	DB12532	Approved; investigational
11	Bimatoprost	−113.888	415.57	DB00905	Approved; investigational
12	Imatinib	−113.493	493.603	DB00619	Approved
13	Dopexamine	−113.319	356.502	DB12313	Approved; investigational
14	Doconexent	−113.105	328.488	DB03756	Approved; investigational

## Data Availability

All data presented in this study are available on request from the corresponding author. The structures of the original data (active set) can be found in the article.

## Supplementary Materials

The following supporting information can be downloaded at: https://www.mdpi.com/article/10.3390/molecules28031296/s1, Figure S1: (A) Superposition of the 18 X-ray structures of Fascin including 11 co-crystallized with ligands, 4 mutant structures, and 3 apo structures. (B) Pairwise RMSD matrix for all Fascin structures calculated for their α carbon atoms; Figure S2: (A) Superposition of the Fascin structures PDB ID: (6I0Z and 6I18). (B) Pairwise RMSD matrix of the backbone of the two proteins. 6I0Z and 6I18 are represented in purple and cyan colors, respectively. (C) Superposition of the Fascin structures’ pockets PDB ID: (6I0Z and 6I18) showing their pairwise RMSD value, (D); Figure S3: Docking poses of the best two ranked compounds (rank 1 and 2) from the bioactives in the binding site of Fascin (PDB:6I18), for (A,B) and (C,D), respectively; Figure S4: pROC-Chemotype plot of the Fascin PDB ID: 6I0Z using the PLANTS docking tool; Figure S5: Docking pose of Lapatinib (DrugBank- cyan sticks) in the Fascin binding site 2 (PDB ID: 6I18), represented by 3D and 2D as (A) and (B), respectively; Figure S6: Docking pose of Fexofenadine (DrugBank- cyan sticks) in the Fascin binding site 2 (PDB ID: 6I18), represented by 3D and 2D as (A) and (B), respectively; Figure S7: Docking pose of compound CP3451 (NANPDB1—orange sticks) in the Fascin binding site 2 (PDB ID: 6I18), represented by 3D and 2D as (A) and (B), respectively; Figure S8: Docking pose of compound CP3270 (NANPDB2—orange sticks) in the Fascin binding site 2 (PDB ID: 6I18), represented by 3D and 2D as (A) and (B); Figure S9: Docking pose of compound CP3756 (orange sticks) in the Fascin binding site 2 (PDB ID: 6I18), represented by 3D and 2D as (A) and (B), respectively; Figure S10: Docking pose of compound CP3407orange sticks) in the binding site 2 of Fascin (PDB ID: 6I18), represented by 3D and 2D as (A) and (B), respectively; Figure S11: Docking pose of compound CP3831(orange sticks) in the binding site 2 of Fascin (PDB ID: 6I18), represented by 3D and 2D as (A) and (B), respectively; Table S1: Fascin structures in the protein data bank (PDB); Table S2: The best enriched 1% of the VS results for NANPDB molecules against Fascin (PDB ID: 6I18); Video S1: Remdesivir -Fascin complex; Video S2: NANPDB3-Fascin complex. References [80,81] are cited in the Supplementary Materials.

## Sample Availability

Samples of the compounds are not available from the authors.

## Abbreviations

DEKOIS	Demanding Evaluation Kits for Objective *In silico* Screening
SBVS	structure-based virtual screening
EF	Enrichment factor
LOA	Level of activity
TOD	Type of data
RMSD	Root mean square deviation
PLANTS	Protein-Ligand ANT System
MOE	Molecular operating environment
SPORES	Structure Protonation and Recognition System
