# Supplementary material for: In Silico Targeting of Fascin Protein for Cancer Therapy: Benchmarking, Virtual Screening and Molecular Dynamics Approaches
Source: Molecules. 2023 Jan 29;28(3):1296. doi: 10.3390/molecules28031296 (PMC9921211; doi:10.3390/molecules28031296)
Supplement: Supplementary file 1 [file molecules-28-01296-s001.zip › Supplementary-Material.pdf]

Supplementary Material

For

In silico Targeting of Fascin protein for Cancer Therapy: Benchmarking, Virtual Screening and Molecular Dynamics Approaches

Heba H. A. Hassan[1], Muhammad I. Ismail[2], Mohammed A.S. Abourehab[3], Frank M. Boeckler[4], Tamer M. Ibrahim[5*], Reem K. Arafa[1, 6*]

[1] Drug Design and Discovery Laboratory, Zewail City of Science and Technology, October Gardens, 6th of October City, 12578 Giza, Egypt
[2] Department of Pharmaceutical Chemistry, Faculty of Pharmacy, The British University in Egypt, Al-Sherouk City, Cairo-Suez Desert Road, 11837 Cairo, Egypt
[3] Department of Pharmaceutics, College of Pharmacy, Umm Al-Qura University, Makkah 21955, Saudi Arabia
[4] Lab for Molecular Design and Pharmaceutical Biophysics, Department of Pharmacy and Biochemistry, Institute of Pharmaceutical Sciences, University of Tübingen, Auf der Morgenstelle 8, 72076 Tübingen, Germany
[5] Department of Pharmaceutical Chemistry, Faculty of Pharmacy, Kafrelsheikh University, Kafrelsheikh 33516, Egypt
[6] Biomedical Sciences Program, University of Science and Technology, Zewail City of Science and Technology, October Gardens, 6th of October City, Giza 12578, Egypt

* Correspondence: Reem Arafa: rkhidr@zewailcity.edu.eg
Tamer M. Ibrahim: Tamer_Mohamad@pharm.kfs.edu.eg, tamer.ibrahim2@gmail.com

* **Correspondence:**
Reem Arafa: rkhidr@zewailcity.edu.eg
Tamer M. Ibrahim: tamer_mohamad@pharm.kfs.edu.eg, tamer.ibrahim2@gmail.com

---

Keywords: Cancer; Fascin; docking; virtual screening (VS); benchmarking; DEKOIS 2.0

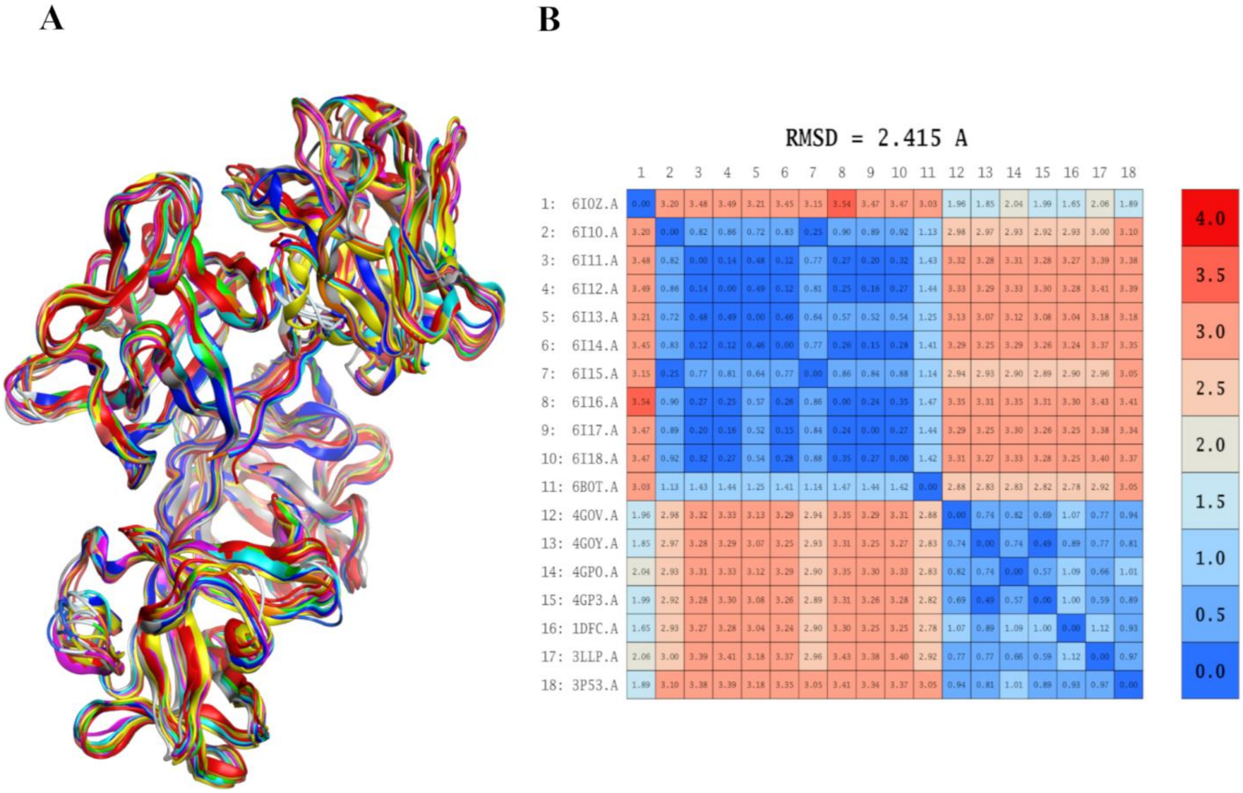

Figure S1. **(A)** Superposition of the 18 X-ray structures of Fascin1 including 11 co-crystallized with ligands, 4 mutant structures and 3 apo structures. **(B)** Pairwise RMSD matrix for all Fascin structures calculated for their α carbon atoms. The color code is assigned randomly.

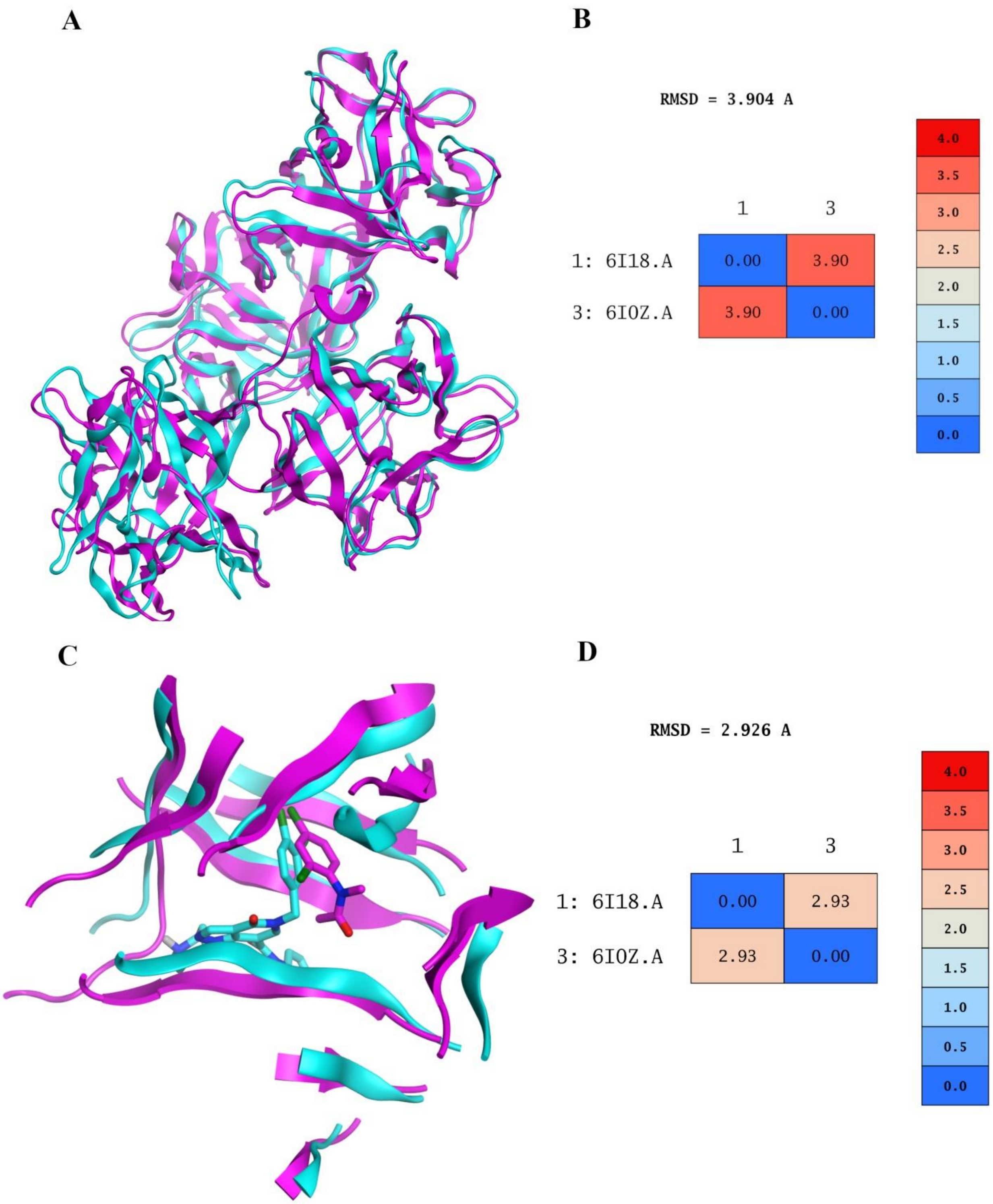

Figure S2. **(A)** Superposition of the Fascin structures PDB ID: (6I0Z and 6I18). **(B)** Pairwise RMSD matrix of the backbone of the two proteins. 6I0Z and 6I18 are represented in purple and cyan colors, respectively. **(C)** Superposition of the Fascin structures' pockets PDB ID: (6I0Z and 6I18) showing their pairwise RMSD value, **(D).**

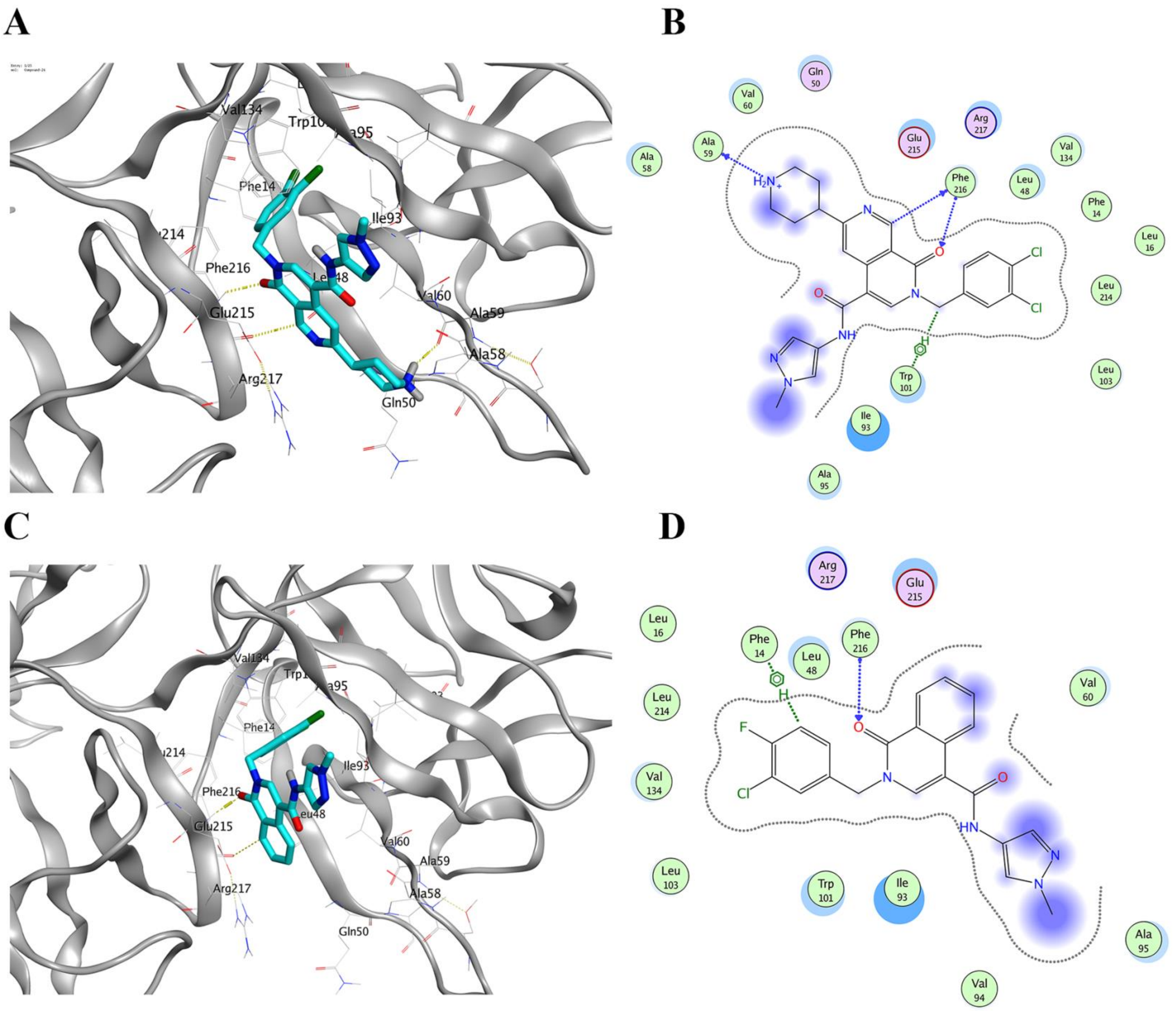

Figure S3. Docking poses of the best two ranked compounds (rank 1 and 2) from the bioactives in the binding site of Fascin (PDB:6I18), for **(A, B)** and **(C, D),** respectively. Yellow dashed lines show favorable interactions. Non-polar hydrogen atoms were ignored for clarification.

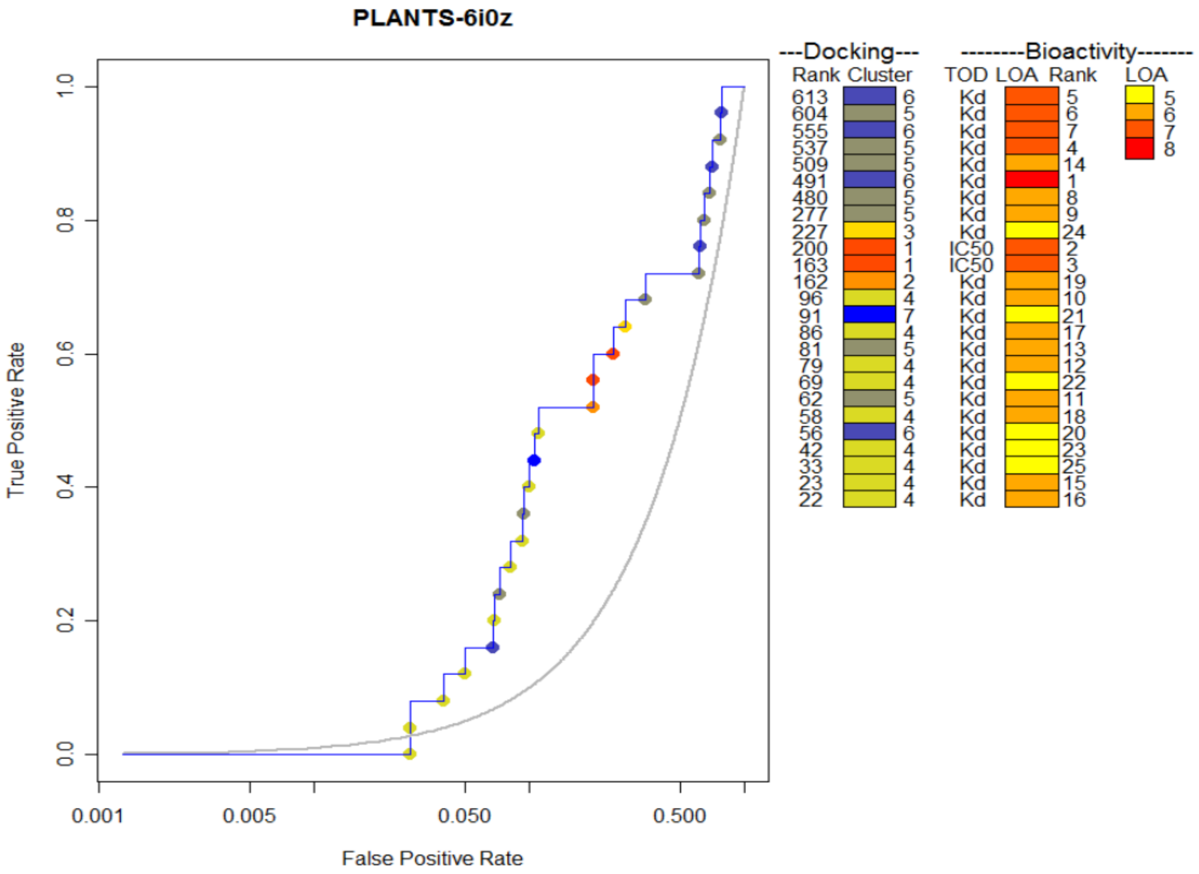

Figure S4: pROC-Chemotype plot of the Fascin PDB ID: 6I0Z using the PLANTS docking tool. The color scheme of the clusters and bioactivity rank is the same of figure 3A.

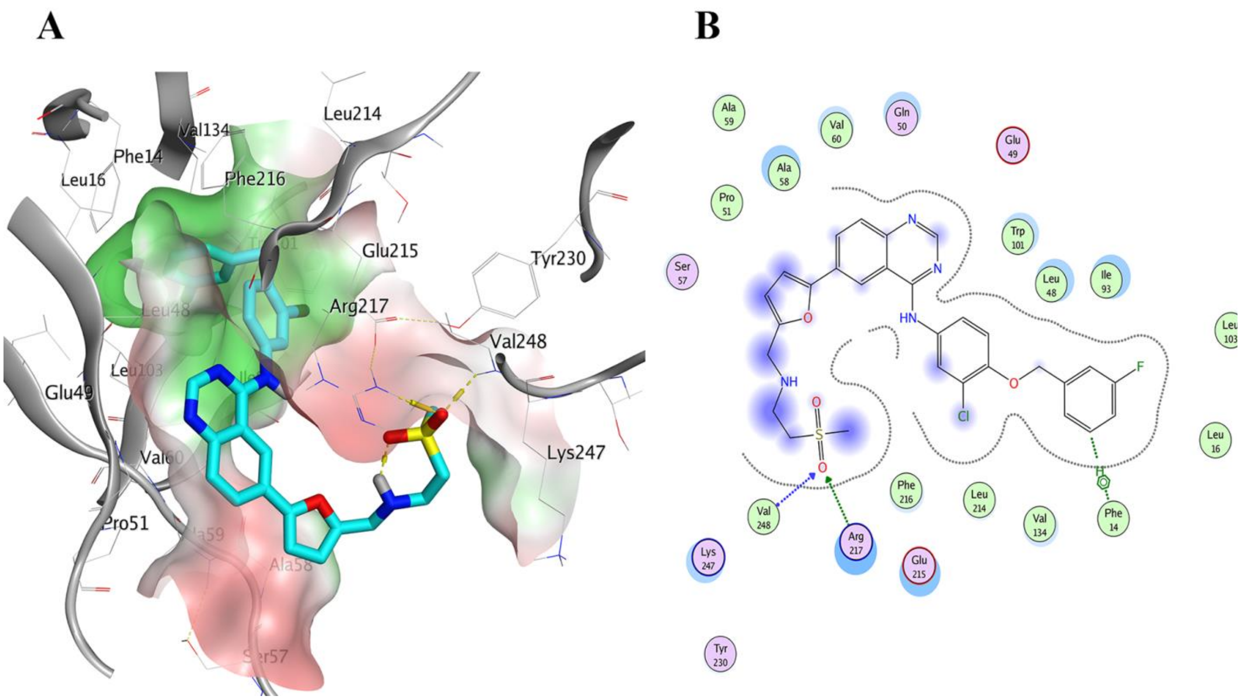

Figure S5. Docking pose of Lapatinib (DrugBank- cyan sticks) in the Fascin binding site 2 (PDB ID: 6I18), represented by 3D and 2D as **(A)** and **(B)**, respectively. Non-polar hydrogen atoms were ignored for clarification. The color scheme is the same as in Figure 4.

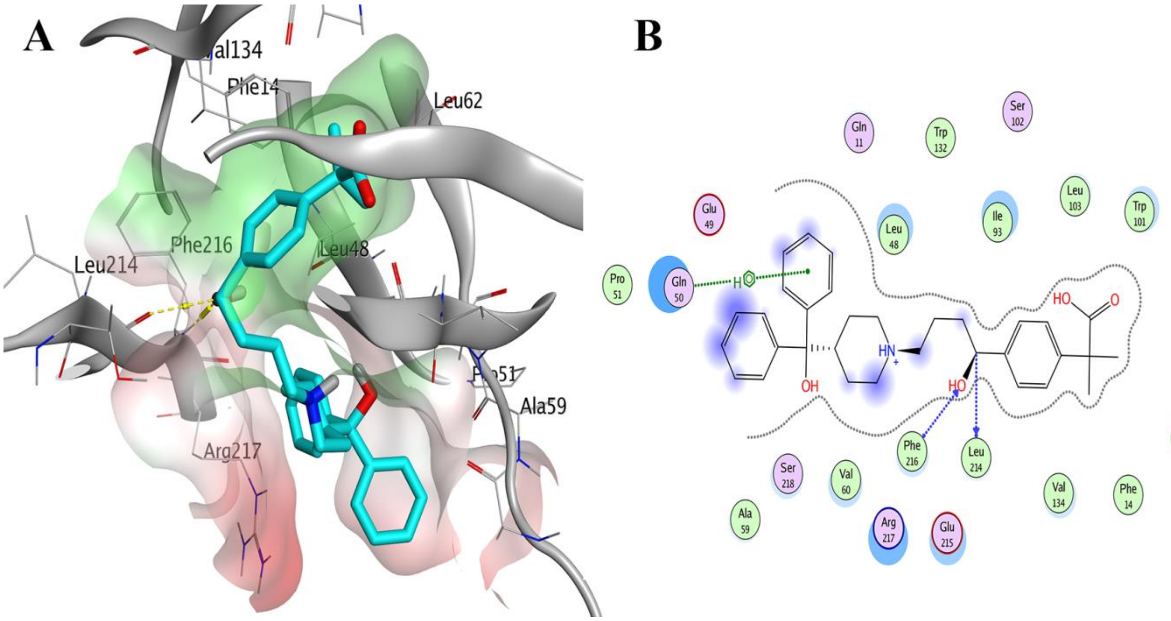

Figure S6. Docking pose of Fexofenadine (DrugBank- cyan sticks) in the Fascin binding site 2 (PDB ID: 6I18), represented by 3D and 2D as (A) and (B), respectively. Non-polar hydrogen atoms were ignored for clarification. The color scheme is the same as in Figure 4.

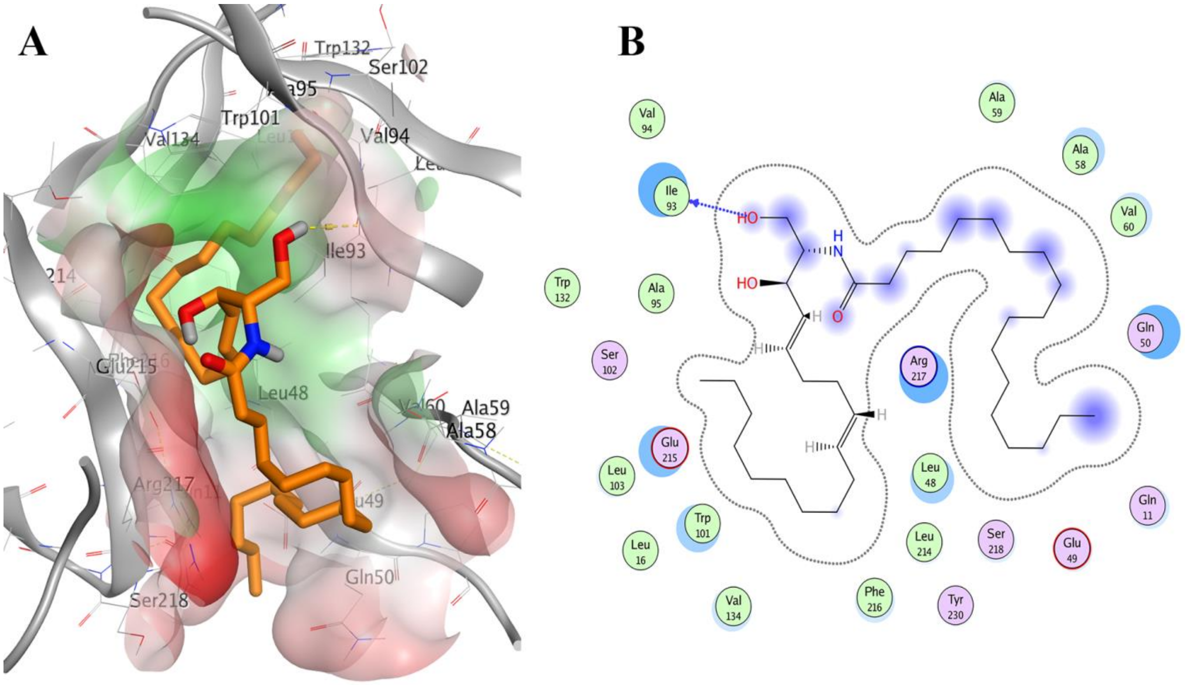

Figure S7. Docking pose of compound CP3451 (NANPDB1 - orange sticks) in the Fascin binding site 2 (PDB ID: 6I18), represented by 3D and 2D as (A) and (B), respectively. Non-polar hydrogen atoms were ignored for clarification. The color scheme is the same as in Figure 4.

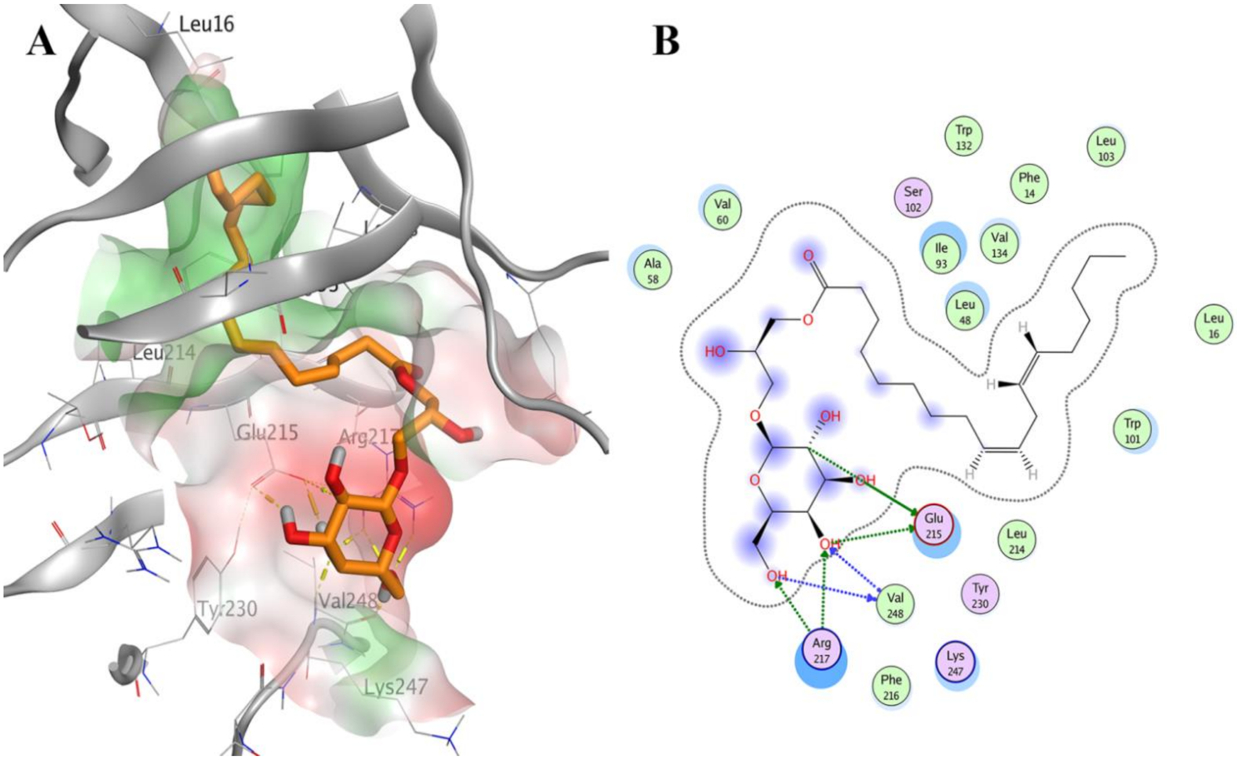

Figure S8. Docking pose of compound CP3270 (NANPDB2 - orange sticks) in the Fascin binding site 2 (PDB ID: 6I18), represented by 3D and 2D as **(A)** and **(B)**, respectively. The color scheme is the same as in Figure 4

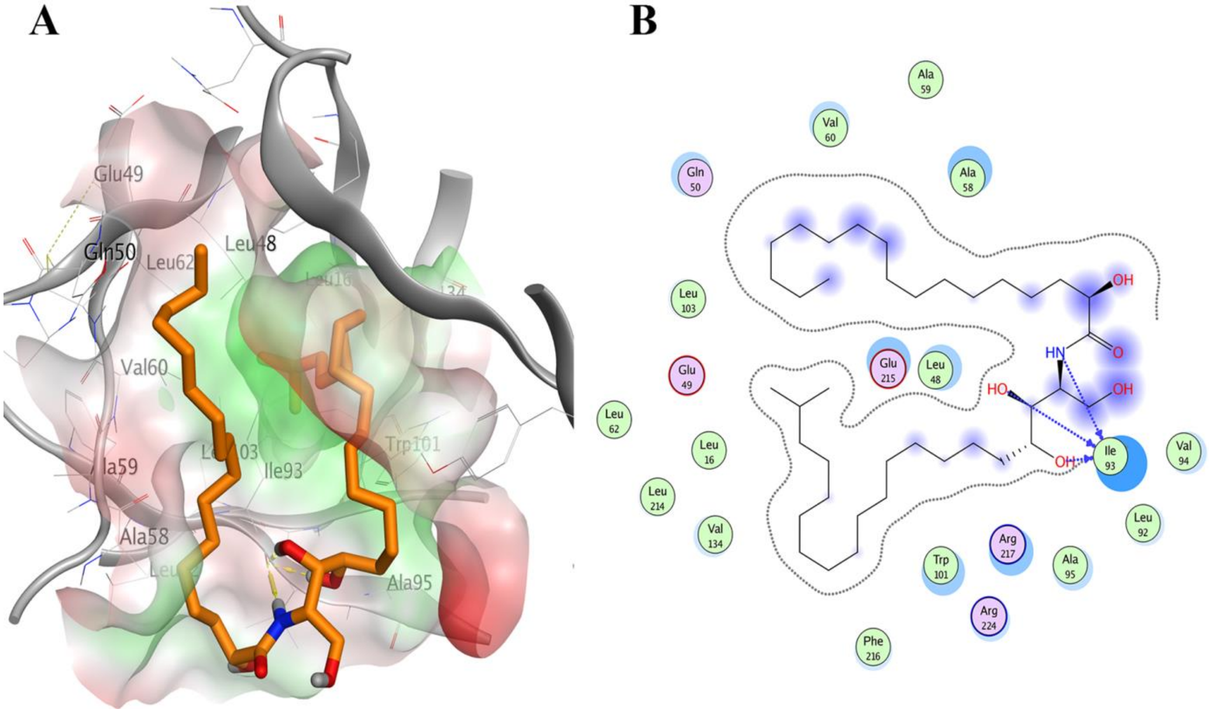

Figure S9. Docking pose of compound CP3756 (orange sticks) in the Fascin binding site 2 (PDB ID: 6I18), represented by 3D and 2D as (A) and (B), respectively. The color scheme is the same as in Figure 4.

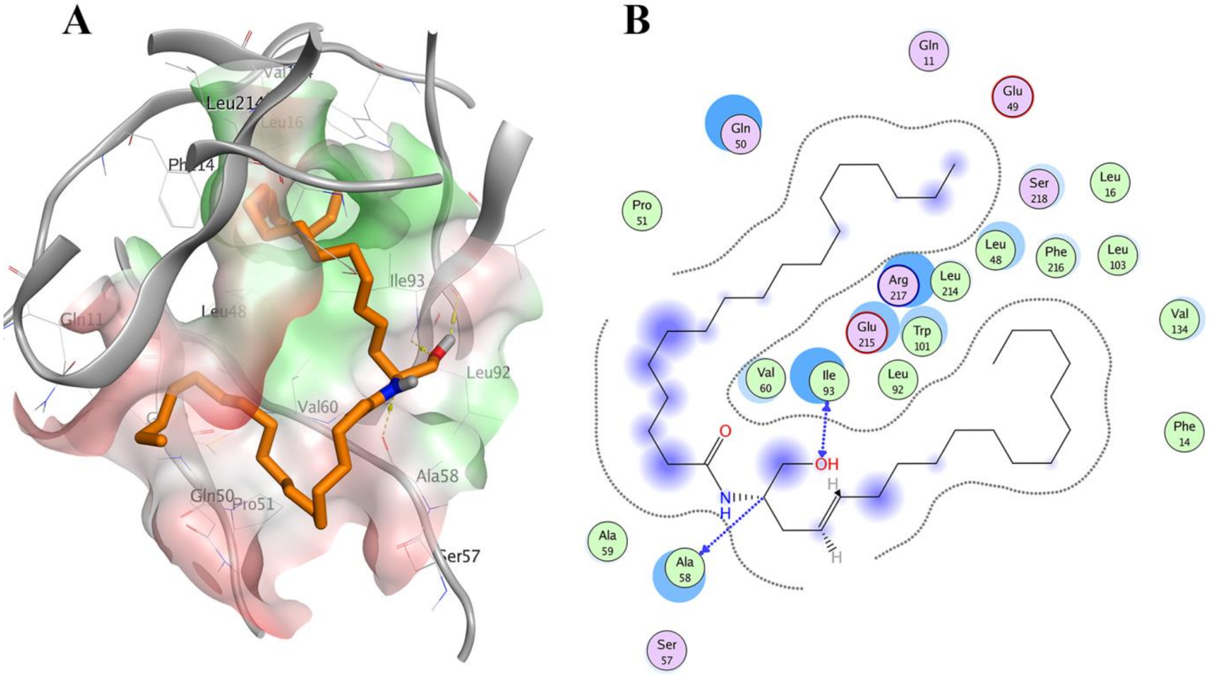

Figure S10. Docking pose of compound CP3407 (orange sticks) in the binding site 2 of Fascin (PDB ID: 6I18), represented by 3D and 2D as (A) and (B), respectively. The color scheme is same as Figure 4.

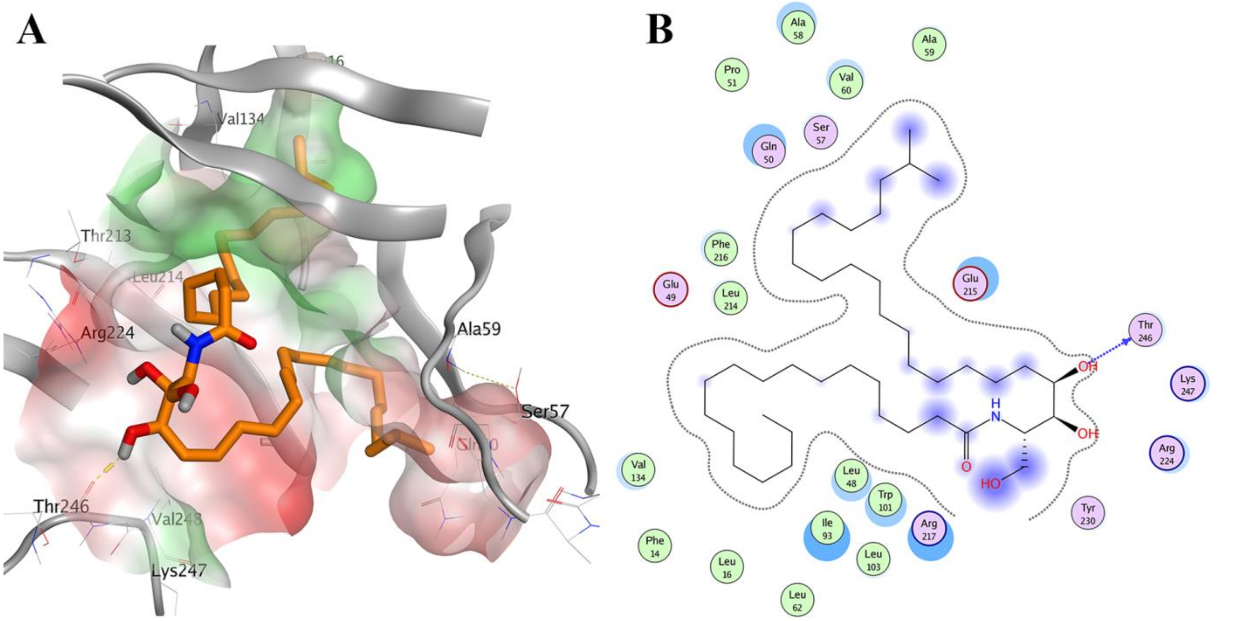

Figure S11. Docking pose of compound CP3831 (orange sticks) in the binding site 2 of Fascin (PDB ID: 6I18), represented by 3D and 2D as (A) and (B), respectively. The color scheme is same as Figure 4.

Table S1: Fascin structures in the protein data bank (PDB)

PDB ID	Structure title	Ligand ID	Binding site	Resolution (A)	Reference
1DFC	Crystal structure of human Fascin, an actin-crosslinking protein.	-	-	2.9	[80]
3LLP	1.8 Angstrom human Fascin 1 crystal structure	-	-	1.8	[21]
3P53	Structure of Fascin	-	-	2	[81]
4GOV	The crystal structure of human Fascin 1 S39D mutant	-	-	2.2	[18]
4GOY	The crystal structure of human Fascin 1 K41A mutant	-	-	2.3	
4GP0	The crystal structure of human Fascin 1 R149A K150A R151A mutant	-	-	2.5	
4GP3	The crystal structure of human Fascin 1 K358A mutant	-	-	2.25	
6I0Z	Crystal structure of Fascin in complex with compound 1	GZQ	2	1.77	[27]
6I10	Crystal structure of Fascin in complex with compound 2	GZK	2	2.1	
6I11	Crystal structure of Fascin in complex with compound 3	H0H	2	1.67	
6I12	Crystal structure of Fascin in complex with compound 5	H08	2	1.65	
6I13	Crystal structure of Fascin in complex with compound 7	H0Q	2	1.79	
6I14	Crystal structure of Fascin in complex with compound 9	GZN	2	1.73	
6I15	Crystal structure of Fascin in complex with compound 11	GZT	2	1.91	
6I16	Crystal structure of Fascin in complex with compound 15	H0B	2	2	
6I17	Crystal structure of Fascin in complex with compound 24	GZW	2	1.56	
6I18	Crystal structure of Fascin in complex with BDP-13176	H0N	2	1.49	
6B0T	Structural Insights into the Induced-fit Inhibition of Fascin by a Small Molecule	C7V	2	2.8	[24]

Table S2: The best enriched 1% of the VS results for NANPDB molecules against Fascin (PDB ID: 6I18).

Docking rank	Structure	Docking score	Name / NANPDB ID	M.wt.
1	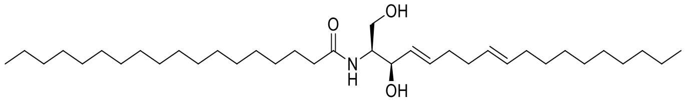	-127.31	2S,3R-4E,8E-2-(octadecanoylamino)-octadeca-4,8-diene-1,3-diol /CP3451	563.94
2	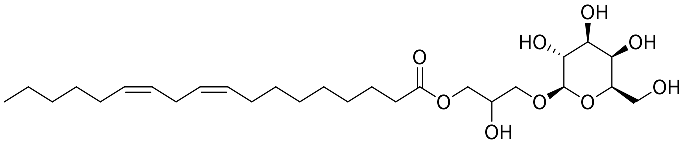	-126.91	1-O-linoleoyl-3-O-beta-D-galactopyranosyl-syn-glycerol /CP3270	516.66
3	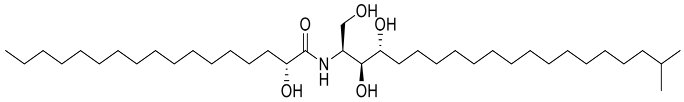	-124.59	Echinoclathriamide /CP3756	628.02
4	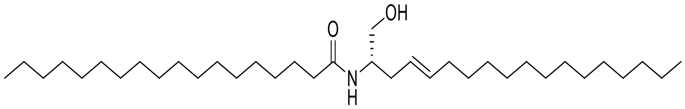	-124.38	2S,3R-4E-2-(octadecanoylamino)-octadec-4-ene-1-ol /CP3407	549.95
5	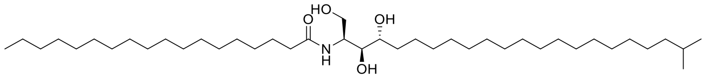	-124.32	Negombata ceramide 1b /CP3831	654.1
6	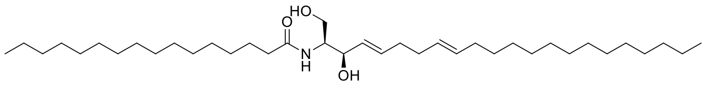	-124.29	2S,3R-4E,8E-2-(hexadecanoylamino)-docosa-4,8-diene-1,3-diol /CP3560	591.99
7	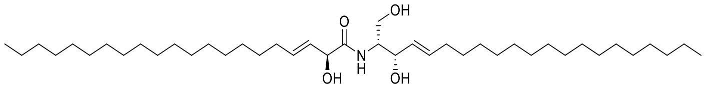	-123.17	Tanacetolide A /CP3852	664.09
8	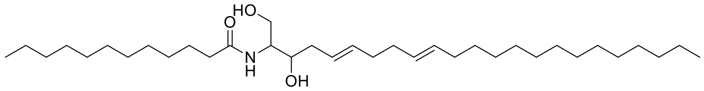	-122.99	Sphinga-4,8 dienine ester /CP3406	549.91
9	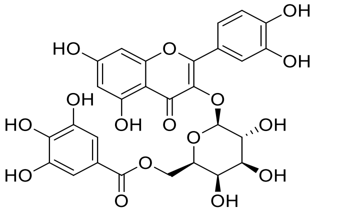	-120.78	Quercetin-3-O-beta-(6"-galloylgalactoside) /CP3685	614.46
10	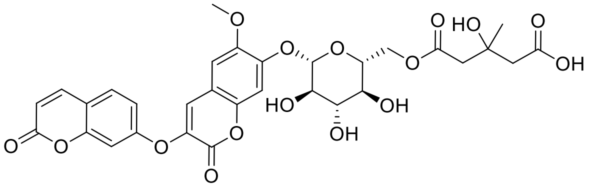	-120.67	Rutarensin /CP3840	657.55

11	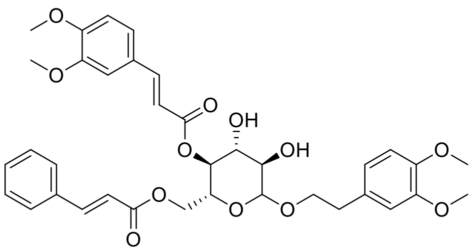	-119.88	1-O-3,4-dimethoxy-phenylethyl-4-O-3,4-dimethoxy cinnamoyl-6-O-cinnamoyl-beta-D-glucopyranose /CP3854	664.69
12	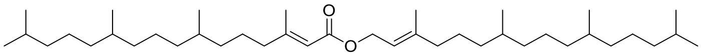	-118.82	Phytyl-3,7,11,15-tetramethyl-2-hexadecanenate /CP3554	589.03
13	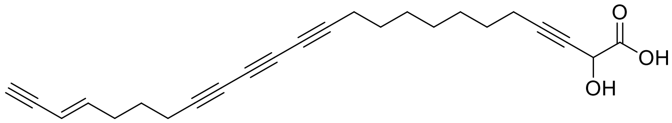	-118.75	Aikupikanyne F /CP1922	361.45
14	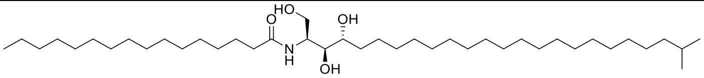	-118.74	Negombata ceramide 1a /CP3832	654.1
15	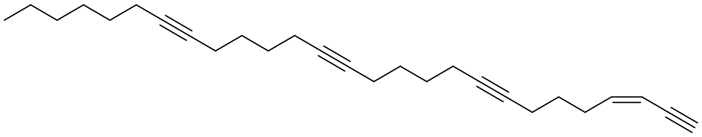	-118.71	C27-polyacetylene /CP1955	362.59
16	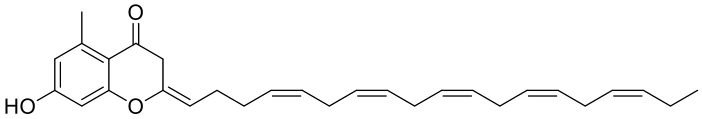	-118.58	2,3-dihydro-7-hydroxy-5-methyl-3-((3Z,6Z,9Z,12Z,15Z)-octadeca-3,6,9,12,15-pentaenyl)chromen-4-one /CP2598	432.59
17	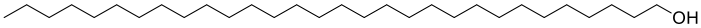	-118.52	Triacontanol/ CP2649	438.81
18	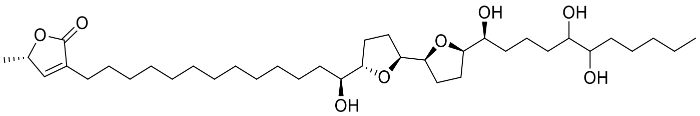	-117.87	Spinescen /CP3792	638.92
19	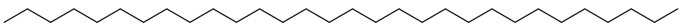	-117.77	Triacontane /CP2480	422.81
20		-117.69	Tritriacontane /CP2920	464.89
21	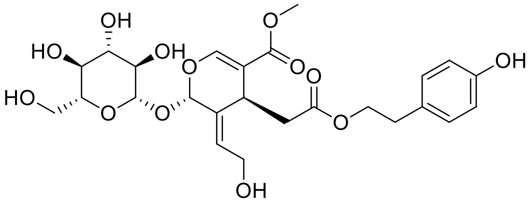	-117.68	10-hydroxyligstroside /CP3371	540.51
22	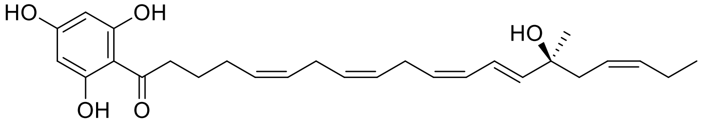	-117.31	(S,5Z,8Z,11Z,13E,17Z)-15-hydroxy-1-(2,4,6-trihydroxyphenyl)-15-methylicosa-5,8,11,13,17-pentaen-1-one/ CP2653	439.56

23	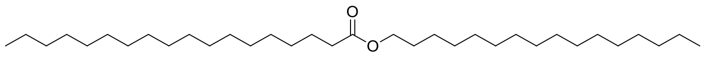	-115.92	Hexadecanyl octadecanoate/ CP3233	508.9
24	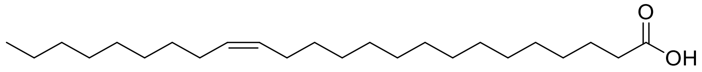	-115.68	Nervonic acid/ CP1991	365.61
25	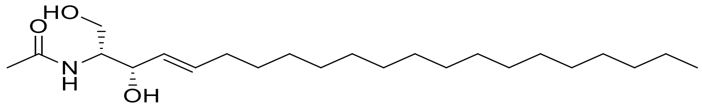	-115.65	Tanacetolide B/ CP2159	383.61
26	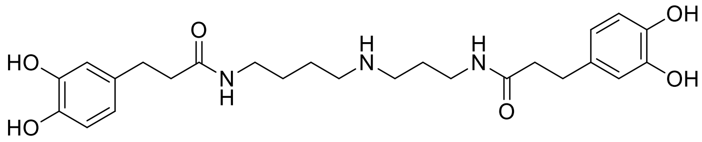	-115.63	N1,N10-di-dihydrocaffeoylspermidine/CP2993	474.57
27	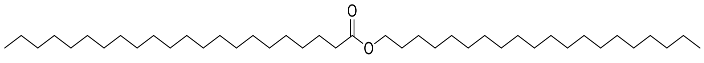	-115.435	Eicosyl docosanoate /CP3699	621.12
28	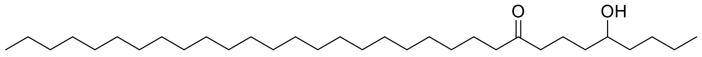	-115.38	5-hydroxydotriacontan-9-one/ CP3059	480.85
29	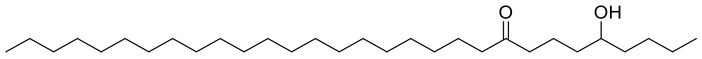	-115.21	5-hydroxytriacontan-9-one/ CP2791	452.79
30	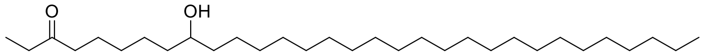	-115.05	23-hydroxyhentriacontan-29-one/ CP2928	466.82
31	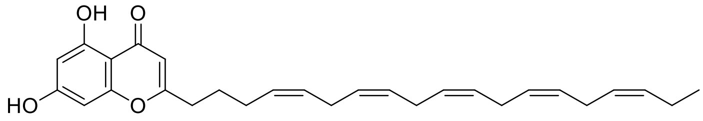	-114.99	2,3-dihydro-5,7-dihydroxy-3-((3Z,6Z,9Z,12Z,15Z)-octadeca-3,6,9,12,15-pentaenyl)chromen-4-one /CP2614	433.56
32	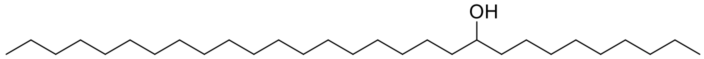	-114.92	Nonacosan-10-ol /CP2503	424.79
33	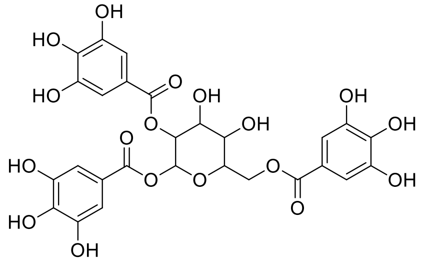	-114.71	1,2,6-tri-O-galloyl-beta-D-glucose/ CP3773	633.44
34	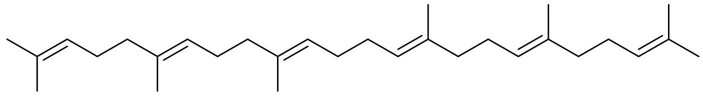	-114.37	Squalene/ CP2381	410.72
35	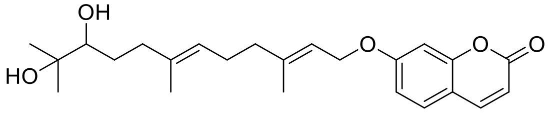	-114.33	Karatavicinol/ CP2307	400.51
36	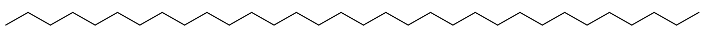	-114.24	Dotriacontane/ CP2784	450.87
37	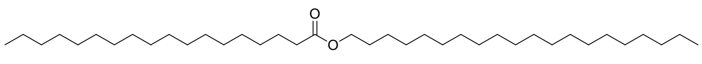	-114.04	Cosanyl octadecanoate/ CP3455	565.01

38	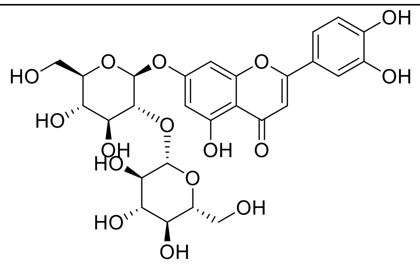	-113.96	Luteolin 7-O-sophoroside/ CP3666	609.51
39		-113.94	Pentatriacontane/ CP3153	492.95

References:

18. Yang, S.; Huang, F.K.; Huang, J.; Chen, S.; Jakoncic, J.; Leo-Macias, A.; Diaz-Avalos, R.; Chen, L.; Zhang, J.J.; Huang, X.Y. Molecular Mechanism of Fascin Function in Filopodial Formation. *J. Biol. Chem.* **2013**, *288*, 274–284, doi:10.1074/JBC.M112.427971.

21. Chen, L.; Yang, S.; Jakoncic, J.; Zhang, J.J.; Huang, X.Y. Migrastatin Analogues Target Fascin to Block Tumour Metastasis. *Nat. 2010 4647291* **2010**, *464*, 1062–1066, doi:10.1038/nature08978.

24. Huang, J.; Dey, R.; Wang, Y.; Jakoncic, J.; Kurinov, I.; Huang, X.Y. Structural Insights into the Induced-Fit Inhibition of Fascin by a Small-Molecule Inhibitor. *J. Mol. Biol.* **2018**, *430*, 1324–1335, doi:10.1016/J.JMB.2018.03.009.

27. Francis, S.; Croft, D.; Schüttelkopf, A.W.; Parry, C.; Pugliese, A.; Cameron, K.; Claydon, S.; Drysdale, M.; Gardner, C.; Gohlke, A.; et al. Structure-Based Design, Synthesis and Biological Evaluation of a Novel Series of Isoquinolone and Pyrazolo[4,3-c]Pyridine Inhibitors of Fascin 1 as Potential Anti-Metastatic Agents. *Bioorg. Med. Chem. Lett.* **2019**, *29*, 1023, doi:10.1016/J.BMCL.2019.01.035.

80. Sedeh, R.S.; Fedorov, A.A.; Fedorov, E. V.; Ono, S.; Matsumura, F.; Almo, S.C.; Bathe, M. Structure, Evolutionary Conservation, and Conformational Dynamics of Homo Sapiens Fascin-1, an F-Actin Crosslinking Protein. *J. Mol. Biol.* **2010**, *400*, 589–604, doi:10.1016/J.JMB.2010.04.043.

81. Jansen, S.; Collins, A.; Yang, C.; Rebowski, G.; Svitkina, T.; Dominguez, R. Mechanism of Actin Filament Bundling by Fascin. *J. Biol. Chem.* **2011**, *286*, 30087–30096, doi:10.1074/JBC.M111.251439.
